# Resveratrol post-transcriptionally regulates pro-inflammatory gene expression via regulation of KSRP RNA binding activity

**DOI:** 10.1093/nar/gku1033

**Published:** 2014-10-28

**Authors:** Franziska Bollmann, Julia Art, Jenny Henke, Katharina Schrick, Verena Besche, Matthias Bros, Huige Li, Daniel Siuda, Norbert Handler, Florian Bauer, Thomas Erker, Felix Behnke, Bettina Mönch, Lorena Härdle, Markus Hoffmann, Ching-Yi Chen, Ulrich Förstermann, Verena M. Dirsch, Oliver Werz, Hartmut Kleinert, Andrea Pautz

**Affiliations:** 1 Department of Pharmacology, Johannes Gutenberg-University Medical Center, Mainz, Germany; 2 Department of Dermatology, Johannes Gutenberg-University Medical Center, Mainz, Germany; 3 Core Facility Lentiviral Transduction Service, Johannes Gutenberg-University Medical Center, Mainz, Germany; 4 Department of Pharmaceutical/Medicinal Chemistry, University of Vienna, Vienna, Austria; 5 Pharmaceutical Institute, University Tuebingen, Tuebingen, Germany; 6 Chair of Pharmaceutical/Medicinal Chemistry, Institute of Pharmacy, Friedrich-Schiller-University, Jena, Germany; 7 pharmazentrum frankfurt/ZAFES, University Hospital, Goethe University Frankfurt, Frankfurt am Main, Germany; 8 Institute of Immunology, Johannes Gutenberg-University Medical Center, Mainz, Germany; 9 Department of Biochemistry & Molecular Genetics, University of Alabama at Birmingham, Birmingham, AL, USA; 10 Department of Pharmacognosy, University of Vienna, Vienna, Austria

## Abstract

Resveratrol shows beneficial effects in inflammation-based diseases like cancer, cardiovascular and chronic inflammatory diseases. Therefore, the molecular mechanisms of the anti-inflammatory resveratrol effects deserve more attention. In human epithelial DLD-1 and monocytic Mono Mac 6 cells resveratrol decreased the expression of iNOS, IL-8 and TNF-α by reducing mRNA stability without inhibition of the promoter activity. Shown by pharmacological and siRNA-mediated inhibition, the observed effects are SIRT1-independent. Target-fishing and drug responsive target stability experiments showed selective binding of resveratrol to the RNA-binding protein KSRP, a central post-transcriptional regulator of pro-inflammatory gene expression. Knockdown of KSRP expression prevented resveratrol-induced mRNA destabilization in human and murine cells. Resveratrol did not change KSRP expression, but immunoprecipitation experiments indicated that resveratrol reduces the p38 MAPK-related inhibitory KSRP threonine phosphorylation, without blocking p38 MAPK activation or activity. Mutation of the p38 MAPK target site in KSRP blocked the resveratrol effect on pro-inflammatory gene expression. In addition, resveratrol incubation enhanced KSRP-exosome interaction, which is important for mRNA degradation. Finally, resveratrol incubation enhanced its intra-cellular binding to the IL-8, iNOS and TNF-α mRNA. Therefore, modulation of KSRP mRNA binding activity and, thereby, enhancement of mRNA degradation seems to be the common denominator of many anti-inflammatory effects of resveratrol.

## INTRODUCTION

Resveratrol is a dietary polyphenol derived from different plant sources like grapes and berries. It possesses an amazing wide spectrum of pharmacological properties resulting in anti-oxidant, anti-diabetic, cardioprotective, anti-cancer, neuroprotective and anti-inflammatory effects (1,2). Thus, resveratrol has beneficial effects in cancer, cardiovascular and chronic inflammatory diseases (3). As inflammation is part of the pathophysiology of all these diseases (4,5), the molecular mechanisms leading to the anti-inflammatory effects of resveratrol deserve more attention.

Beside modest anti-oxidant properties (2), resveratrol has been shown to modulate signal transduction pathways like the p38 MAPK (6) or AMPK pathway (7) and the activity of different pro-inflammatory transcription factors like nuclear factor-kB (NF-κB) (8) or signaltransducer and activator of transcription-1a (STAT-1α) (9). Many of these signaling pathways or transcription factors appear to be redox-regulated and, thus, some of these cellular resveratrol effects have been proposed to be a consequence of its anti-oxidant properties (10). Some data show that resveratrol modulates signaling pathways redox-independently (11).

The anti-inflammatory properties of resveratrol were mostly attributed to its inhibitory effects on key pro-inflammatory transcription factors like NF-κB (12) or STAT-1α (9). However, recent data suggest that dysregulated post-transcriptional regulation of pro-inflammatory gene expression plays a central role in the onset and maintenance of chronic inflammatory diseases (13–15). Only a few examples indicate post-transcriptional effects of resveratrol. It stabilizes eNOS mRNA in human EA.hy 926 endothelial cells (16) and reduces the mRNA stability of CYP1A1 in human T47D breast cancer cells (17). In human Jurkat T-cells resveratrol was found to modulate the cellular localization of the RNA binding protein HuR (18) and thereby suppresses tumor necrosis factor-α (TNF-α) expression post-transcriptionally. Exact mechanisms of the post-transcriptional effects of resveratrol are completely unknown.

Several effects of resveratrol were attributed to its ability of activating the histone deacetylase sirtuin 1 (SIRT1) (12). Meanwhile, studies questioned the role of SIRT1 as a direct binding partner of resveratrol (19). In direct binding assays only a few resveratrol target proteins like the hetero-dimeric alphaVbeta3 integrin, the quinone reductase NAD(P)H dehydrogenase, quinone 2, the glutathione sulfotransferase-p1 and the estrogen receptor-beta have been identified so far (20–22). Recently, by receptor binding studies, also different phosphodiesterases (PDE-1, -3 and -4) have been detected as direct resveratrol targets (7).

The KH-type splicing regulatory protein (KSRP) is an important regulator of multiple inherently instable mRNAs mostly coding for pro-inflammatory mediators like TNF-α (23), interleukin-8 (IL-8) (24) or inducible nitric oxide synthase (iNOS) (25). By binding to AU-rich elements (AREs) in the 3′-untranslated region (3′-UTR), KSRP recruits enzymes involved in the 5′- and 3′-mRNA decay (26). Thus, KSRP seems to be a central component of the ARE-mediated decay of mRNAs. Protein phosphorylation at threonine (T692 by p38 MAPK) or serine (S132, S274, S670 by ataxia telangiectasia mutated (ATM)-kinase and S193 by PI3K-AKT) residues has been shown to regulate KSRP activity (27). KSRP may also regulate pro-inflammatory gene expression in an indirect way as it promotes the maturation of a specific subset of microRNAs (miRNAs) (26), including miR-155 (28).

In this study incubation of human DLD-1 or Mono Mac 6 cells with resveratrol markedly reduced cytokine-induced expression of IL-8, iNOS and TNF-α. This inhibition resulted from resveratrol-mediated reduction of mRNA stability and was independent of SIRT1. In a target-fishing approach with resveratrol as bait and peripheral blood mononuclear cell (PBMC) as target source, we identified KSRP as a major, direct binding target of resveratrol. In drug responsive target stability experiments, resveratrol enhanced KSRP protein stability. KSRP is an important regulator of the mRNA stability of pro-inflammatory genes and is also involved in miRNA maturation. Accordingly, downregulation of KSRP expression by siRNA diminished the post-transcriptional effects of resveratrol on pro-inflammatory gene expression in human DLD-1 cells. Also in primary cells isolated from mice with inactivated KSRP gene, the inhibitory effect of resveratrol on pro-inflammatory gene expression was markedly diminished. The mRNA binding activity of KSRP has has been shown to be regulated by ATM-kinase-, PI3K-AKT- and p38 MAPK-mediated protein phosphorylation (26). Only inhibition of p38 MAPK reduced cytokine-induced iNOS expression. Therefore, we analyzed the effects of resveratrol on p38 MAPK-mediated KSRP phosphorylation. We observed reduction of the inhibitory p38 MAPK-dependent phosphorylation of threonine 692 in KSRP after resveratrol treatment without effects of p38 MAPK activation or activity. Accordingly, mutation of threonine 692 to alanine in the p38 MAPK target site of KSRP markedly diminished the resveratrol effect on pro-inflammatory gene expression in DLD-1 cells. Immunoprecipitation experiments showed enhanced KSRP-exosome interaction after resveratrol treatment. Finally, analysis of the intra-cellular binding of KSRP to the IL-8-, iNOS- and TNF-α mRNA showed enhanced interaction of KSRP with these mRNAs in resveratrol-treated cells. Both results explain the reduced mRNA stability after resveratrol treatment.

## MATERIALS AND METHODS

### Materials

Materials used are described in Supplementary Data.

### Cell culture, cytokine and resveratrol treatment and RNA isolation

Human wild-type epithelial colon carcinoma DLD-1 cells (ATCC, #CCL-221), DLD-1-16kb-iNOS-promoter cells (29), DLD-1-TNF-α-prom-, DLD-1-IL-8-prom-, DLD-1-GAS- or -NF-κB-promoter cells, DLD-1-CDH-siLuc, DLD-1-CDH-siKSRP, DLD-1-pGIPZ-CO, DLD-1-pGIPZ-siKSRP cells (all generated as described below) and human epithelial kidney HEK 293T cells (ATCC, #CRL-11268) were cultivated in Dulbecco's modified Eagle's medium (DMEM) with 10% inactivated fetal bovine serum (FCS), 2 mM L-glutamine, 100 U/ml penicillin and 100 μg/ml streptomycin, as well as 50 μM β-mercaptoethanol (only HEK 293 T cells). Human monocytic Mono Mac 6 cells (DSMZ #ACC 124) were cultivated in RPMI 1640 + 10% inactivated FCS, 2 mM L-glutamine, 100 U/ml penicillin, 100 μg/ml streptomycin and 1 mM sodium pyruvate. Sixteen hours before cytokine induction, cells were washed with phosphate buffered saline (PBS) and incubated with DMEM or RPMI containing 2 mM L-glutamine in the presence of 0.5% FCS (Mono Mac 6) or absence of FCS and phenol red. Afterwards, cells were pre-incubated with the indicated concentrations of resveratrol or the different kinase inhibitors for 1 h. iNOS (only DLD-1), IL-8 and TNF-α expression was induced using a cytokine mixture (CM) containing IFN-γ (100 U/ml), IL-1β (50 U/ml) and TNF-α (10 ng/ml) for different time periods depending on the experiment. Then cells were processed for RNA isolation by guanidinium thiocyanate/phenol/chloroform extraction as described (25) or for protein extraction and luciferase assays as described below.

### Animals

All mice were housed in accordance with standard animal care requirements and maintained under specified pathogen-free conditions on a 12/12-h light/dark circle. Water and food were given *ad libitum*. The animal studies were approved by the ethical board and were performed in accordance with the German animal protection law and the guidelines for the use of experimental animals as stipulated by the Guide of Care and Use of Laboratory Animals of the National Institutes of Health. Mice were euthanized by *i.p.* injection of 700 μl Pentobarbital solution (1% Pentobarbital in PBS).

KSRP^+/−^ mice (30) had a C57BL/6 background. Experimental KSRP^−/−^- and KSRP^+/+^ animals were obtained by mating KSRP^+/−^ animals. Genotyping of the animals was performed by polymerase chain reaction, using primers that span the region of the wild-type gene flanked by loxP sites deleted by the Cre recombinase (30). The following oligonucleotides (obtained from Sigma-Aldrich; Hamburg, Germany) were used for genotyping the KSRP-locus: KSRP-wt-for GCGGGGAGAATGTGAAGG, KSRP-ko-for CTCCGCCTCCTCAGCTTG and KSRP-wt/ko-rev GAGGCCCCTGGTTGAAGG.

### Analysis of CXCL-1, iNOS and TNF-α mRNA expression in peritoneal cells of KSRP^−/−^ or KSRP^+/+^ animals

To analyze the CXCL-1, iNOS and TNF-α mRNA expression in primary cells of KSRP^−/−^ or KSRP^+/+^ animals, we isolated peritoneal cells as described by Ray *et al.* (31). Adherent cells (mostly monocytes/macrophages) were pre-incubated with or without 30 μM resveratrol and treated with LPS (2 μg/ml) and IFN-γ (100 U/ml) to induce pro-inflammatory mRNA expression. After 2–6 h cells were lyzed and the expression of CXCL-1, iNOS, TNF-α and Glyceraldehyde 3-phosphate dehydrogenase (GAPDH) mRNA was measured by quantitative reverse transcription-polymerase chain reaction (qRT-PCR). CXCL-1, iNOS and TNF-α mRNA expression was normalized to GAPDH mRNA expression.

### Isolation of PBMC from human blood and preparation of cell lysates

Isolation of PBMC and extract preparation were performed as described in Supplementary Data.

### Drug responsive target stability

Human KSRP (500 μg/ml; expressed in bacteria as described in (25)) was solubilized in TNC buffer (50 mM Tris pH 8.0, 50 mM NaCl, 10 mM CaCl_2_). Note that 15 μg KSRP was pre-treated for 15 min at 37°C with 30 μM resveratrol or vehicle (0.7% DMSO), respectively. Pre-treated protein samples were digested with pronase (0.15 μg, corresponding to 1:100 pronase/protein ratio) for 60 min at 37°C. Digestion was stopped by addition of 2× sodium dodecyl sulphate-polyacrylamide gel electrophoresis (SDS-PAGE) sample loading buffer (100 mM Tris-HCl, pH 6.8, 4% SDS, 20% glycerol, 2% 2-mercaptoethanol, 25 mM ethylenediaminetetraacetic acid (EDTA), 0.04% bromophenol blue) and heated to 95°C for 5 min.

Digested protein samples were separated on 10% polyacrylamide gels by SDS-PAGE and transferred to nitrocellulose membranes. Membranes were incubated with mouse anti-KSRP antibody and goat anti-mouse secondary antibody. Proteins were detected and analyzed with the Odyssey infrared imaging system (LI-COR Biosciences). For densitometry analysis, integrated intensities of respective bands were normalized to the signals of matching undigested controls (e.g. resveratrol digested/resveratrol undigested).

### Establishment of cell lines expressing a mutated KSRP or anti-KSRP shRNAs by lentiviral transduction

The generation of the lentiviral-transduced cells was performed as described in Supplementary Data.

### siRNA-mediated downregulation of SIRT1 expression

Downregulation of SIRT1 expression in DLD-1 cells was performed as described in Supplementary Data.

### qRT-PCR

Gene expression was quantified in a two-step real-time RT-PCR using either Taqman probes (25) or SYBR Green as described before (32). The sequences of the oligonucleotides used as sense and antisense primers as well as Taqman hybridization probes are provided in Supplementary Data. To calculate the relative mRNA expression rates the 2^ΔΔ*C*(*T*)^ method (33) was used. According to this method the *C*(*T*) values for CXCL-1, iNOS, IL-8, SIRT1 or TNF-α mRNA in each sample were normalized to the *C(T*) values of GAPDH mRNA in the same sample. Then, the values of CM-treated cell samples or untreated mice were set to 100% and the percentage of CXCL-1, iNOS, IL-8, SIRT1 or TNF-α mRNA expression was calculated.

### Western blot experiments

To study the expression of KSRP, phospho-threonine-modified KSRP, p38 MAPK, phospho-p38 MAPK, ATF2, phospho-ATF2, iNOS, SIRT1, GAPDH, PM-Scl or β-tubulin in DLD-1 or murine cells, total cellular protein (10–50 μg protein) was separated on SDS polyacrylamide gels and transferred to nitrocellulose membranes by semi-dry electroblotting. All further steps were performed as described previously (25). For detection of iNOS, KSRP, phospho-threonine-modified KSRP, p38 MAPK, phospho-p38MAPK, ATF2, phospho-ATF2, SIRT1, PM-Scl, GAPDH and β-tubulin, the antibodies listed in Supplementary Data were used. The immunoreactive proteins on the blots were visualized by the enhanced chemiluminescence detection system.

### Immunoprecipitation

For immunoprecipitation, cell extracts were digested for 30 min at 30°C with RNase A (40 μg) and RNAse T1 (500 U). Then these extracts were pre-incubated with protein A/G-agarose beads in RIPA buffer (50 mM Tris-HCl, pH 7.4, 150 mM NaCl, 2 mM EDTA, 10% glycerol, 1% NP40, 1× complete EDTA-free protease and phosphatase inhibitor cocktail) for 1 h at 4°C. These pre-cleared extracts were incubated with a polyclonal KSRP antibody overnight at 4°C. Subsequently, protein-antibody complexes were captured by incubation with protein A/G-agarose beads for 5 h at 4°C. Beads were washed with RIPA buffer and co-immunoprecipitated proteins were analyzed by western blotting.

### Immunoprecipitation-qRT-PCR assay

Determination of intra-cellular protein-RNA interactions was performed using immunoprecipitation-qRT-PCR analyses as described in Supplementary Data.

### Analysis of resveratrol effects on the human iNOS, IL-8 or TNF-α promoter activity and artificial GAS- or NF-κB-dependent promoters

The promoter analyses were performed as described in supplementary data.

### Analysis of resveratrol effects on 3′-UTR activity

The analysis of the effects of resveratrol on the human iNOS-3′-UTR and SHIP-3′-UTR activity were performed as described in Supplementary Data.

### iNOS, IL-8 or TNF-α mRNA stability measurements

To examine the influence of resveratrol on iNOS, IL-8 or TNF-α mRNA stability in DLD-1, DLD-1-siKSRP, DLD-1-siLUC or Mono Mac 6 (only IL-8 and TNF-α) cells, iNOS, IL-8 or TNF-α mRNA expression was induced by CM-treatment for 1 or 4 h as indicated. Note that 30 μM resveratrol was added. Thirty minutes later 6-dichloro-1-ribofuranosylbenzimidazole (DRB) (25 μg/ml) was added to stop RNA Polymerase II-dependent transcription. After 10, 20, 30, 45 min, or 2, 4 and 6 h the expression of iNOS, IL-8, TNF-α and GAPDH (for normalization) mRNA was measured. iNOS, IL-8 or TNF-α mRNA expression was normalized to GAPDH mRNA expression. The relative amounts of iNOS, IL-8 or TNF-α mRNA at 0 h DRB were set to 100%. Curve fittings of the resulting DRB time curves were performed by non-linear regression.

### Protein-fishing assays

The protein-fishing analyses were performed as described in Supplementary Data.

### Statistics

Data represent means ± SEM. Statistical differences were determined by factorial analysis of variance followed by ‘Tukey's’ or ‘Dunnett's’ multiple comparison test. In the case of two means, classical *t*-test analyses were used. To compare the mRNA stability, two-way ANOVA analysis followed by Bonferroni's multiple comparisons test was performed. All statistical analyses were performed using Graphpad Prism 6.0.

## RESULTS

### Resveratrol inhibits pro-inflammatory gene expression in human DLD-1 cells

The anti-inflammatory actions of resveratrol are attributed to its inhibitory effects on key pro-inflammatory transcription factors like NF-κB (12) or STAT-1α (9). Recent data indicate that dysregulated post-transcriptional regulation of pro-inflammatory gene expression plays a major role in the pathogenesis and chronification of inflammatory diseases (13–15).

**Figure 1.**
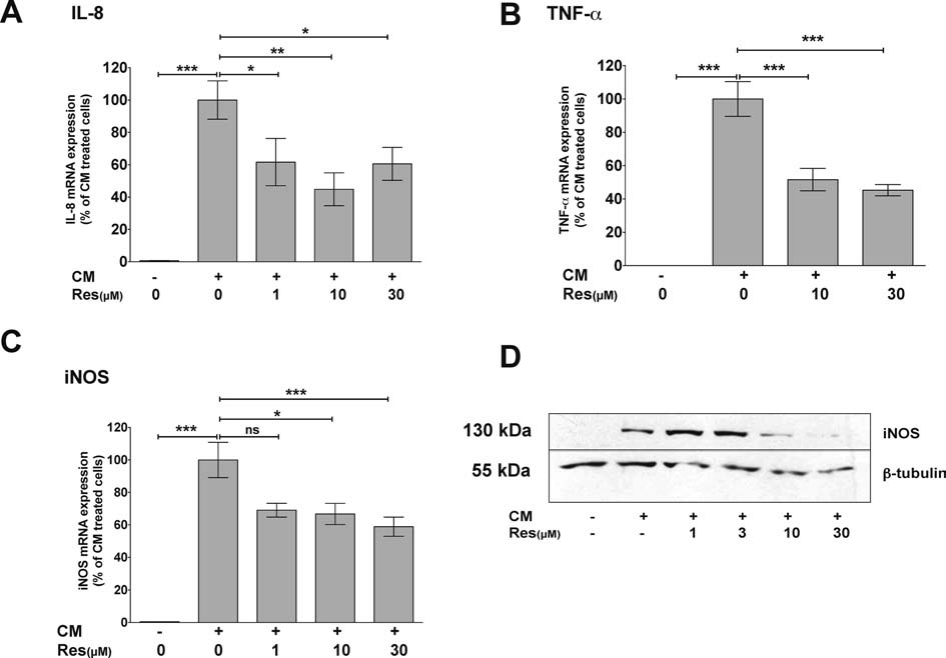
Resveratrol reduces IL-8, TNF-α and iNOS mRNA, as well as iNOS protein expression in human epithelial DLD-1 cells. (A–C) DLD-1 cells were pre-incubated with resveratrol (Res; 1–30 μM) for 1 h and then treated with a CM containing IFN-γ (100 U/ml), IL-1β (50 U/ml) and TNF-α (10 ng/ml) for additional 2 h (IL-8 and TNF-α) or 6 h (iNOS). RNA was isolated and analyzed for IL-8 (A), TNF-α (B), iNOS (C) and GAPDH (for normalization) mRNA expression by qRT-PCR. Data shown are mean ± SEM of *n* = 8–10 qRT-PCR analyses. The CM-induced mRNA expression was set to 100% (***P < 0.001; **P < 0.01; *P < 0.05; ns, not significant versus CM-treated DLD-1 cells; one-way ANOVA). (D) DLD-1 cells were pre-incubated with resveratrol (Res; 1–30 μM) for 1 h and then treated with CM for additional 6 h. Proteins were isolated and analyzed for iNOS and β-tubulin (for normalization) protein expression by western blot using specific antibodies. Shown is one (of four) representative western blots.

Therefore, we investigated the effect of resveratrol on the cytokine-induced expression of three important pro-inflammatory marker genes, IL-8, iNOS and TNF-α in human epithelial DLD-1 cells. The cells were pre-treated with resveratrol (**Res**; 1–30 μM) for 1 h and incubated with a CM (IFN-γ, IL-1β and TNF-α) for 2 (TNF-α and IL-8) or 6 h (iNOS). Afterwards, we analyzed IL-8, TNF-α and iNOS mRNA expression by qRT-PCR and iNOS protein expression by western blot. As shown in Figure 1, resveratrol inhibited the CM-induced expression of all three mRNAs and iNOS protein expression.

### Resveratrol destabilizes iNOS, IL-8 and TNF-α mRNA in DLD-1 cells

**Figure 2.**
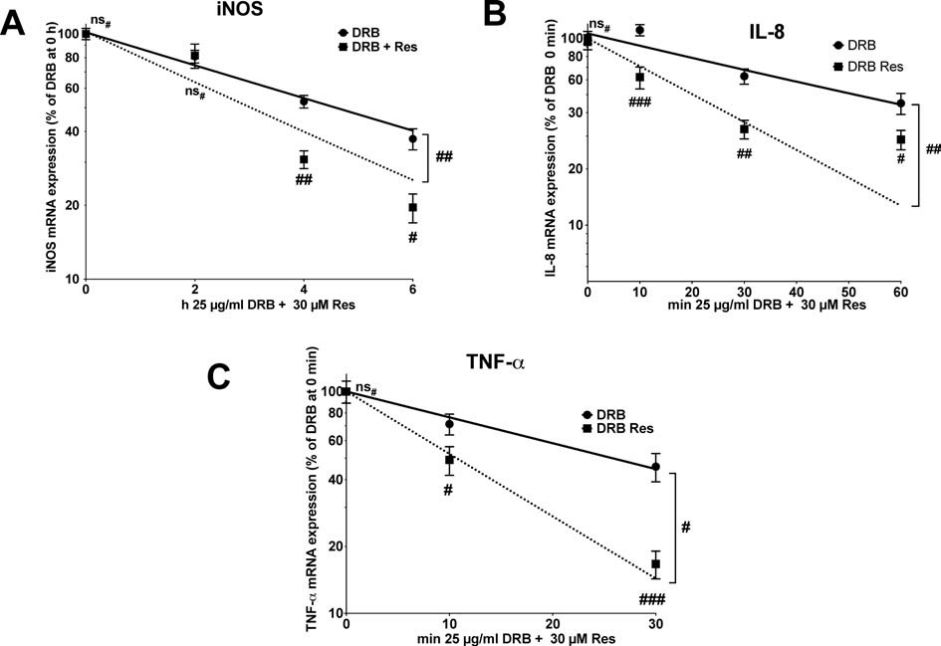
Resveratrol reduces the stability of iNOS, IL-8 and TNF-α mRNA in human epithelial DLD-1 cells. DLD1 cells were treated with CM for 1 or 4 h. Afterwards, 30 μM resveratrol was added. Thirty minutes later the cells were treated with DRB (25 μg/ml) to stop RNA Polymerase II-dependent transcription. After 10, 30, 60 min, or 2, 4 and 6 h the expression of iNOS (A), IL-8 (B), TNF-α (C) and GAPDH (for normalization) mRNA was measured. iNOS, IL-8 or TNF-α mRNA expression was normalized to GAPDH mRNA expression. The relative iNOS, IL-8 and TNF-α mRNA expression after 1 or 4 h CM was set to 100%. Shown are the mean ± SEM of *n* = 10–12 analyses (###*P* < 0.001; ##*P* < 0.01; #*P* < 0.05; ns_#_, not significant versus cells not treated with resveratrol; two-way ANOVA). The half-lifes of the respective mRNAs are iNOS: CM 4.39 ± 0.54 h, CM + Res 2.86 ± 0.55 h; IL-8: CM 42.89 ± 11.11 min, CM + Res 20.16 ± 5.67 min; TNF-α: CM 25.80 ± 9.99 min, CM + Res 10.60 ± 4.75 min.

We observed no inhibitory effects of resveratrol on human iNOS, IL-8 or TNF-α promoter activity (see Supplementary Figure S1), but an inhibitory effect of resveratrol on iNOS-3′-UTR-dependent luciferase expression (see Supplementary Figure S2). Therefore, we measured the effects of resveratrol on iNOS, IL-8 or TNF-α mRNA stability in DLD-1 cells. We pre-incubated cells with CM and resveratrol before adding DRB to stop RNA-Polymerase II-dependent transcription. Residual iNOS, IL-8 or TNF-α mRNA amounts were measured by qRT-PCR at the time points indicated. In DLD-1 cells treated with resveratrol (30 μM) the stability of the iNOS, IL-8 and TNF-α mRNA was significantly reduced in comparison to DLD-1 cells not incubated with resveratrol (see Figure 2). These data indicate that the effects of resveratrol on human iNOS, IL-8 and TNF-α expression are primarily mediated on the post-transcriptional level *via* destabilization of the mRNAs.

### Resveratrol inhibits pro-inflammatory gene expression in human Mono Mac 6 cells by reduction of mRNA stability

**Figure 3.**
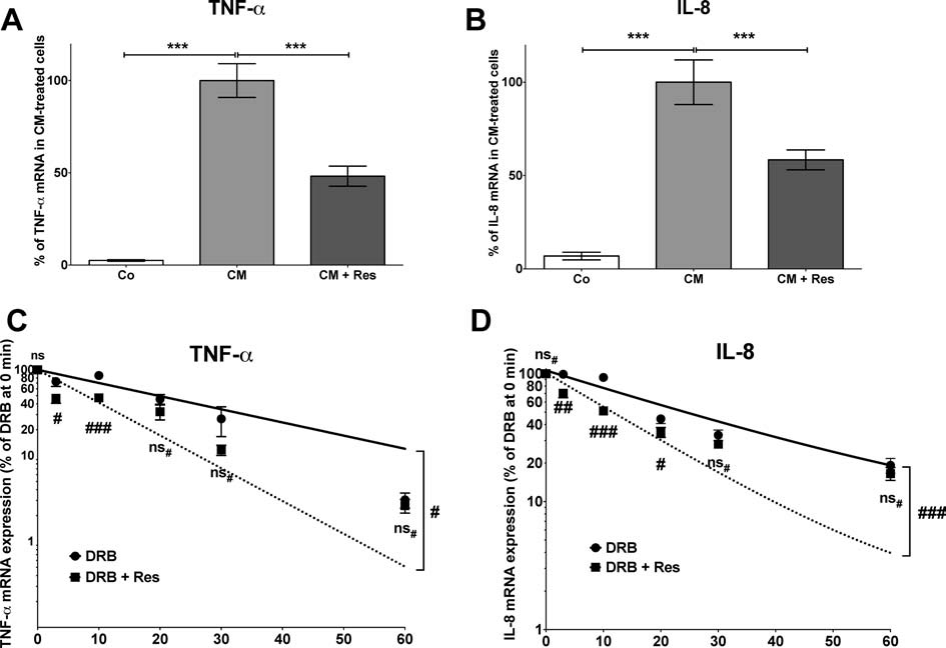
Resveratrol reduces the stability of IL-8 and TNF-α mRNA in human monocytic Mono Mac 6 cells. (A and B) Mono Mac 6 cells were pre-incubated with 30 μM resveratrol (Res) for 1 h and then treated with CM for additional 2 h. RNA was isolated and analyzed for TNF-α (A), IL-8 (B) and GAPDH (for normalization) mRNA expression by qRT-PCR. Data shown are mean ± SEM of *n* = 8–10 qRT-PCR analyses. The CM-induced mRNA expression was set to 100% (***P < 0.001 versus CM-treated Mono Mac 6 cells; one-way ANOVA). (C and D) Mono Mac 6 cells were treated with CM for 2 h. Note that 30 μM resveratrol was added and 30 min later cells were treated with DRB (25 μg/ml) to stop RNA Polymerase II-dependent transcription. After 10, 30 and 60 min the expression of TNF-α (A), IL-8 (B) and GAPDH (for normalization) mRNA was measured. IL-8 or TNF-α mRNA expression was normalized to GAPDH mRNA expression. The relative IL-8 or TNF-α mRNA expression after 2 h CM was set to 100%. Shown are the mean ± SEM of *n* = 10–12 analyses (###*P* < 0.001; ##*P* < 0.01; #*P* < 0.05; ns_#_, not significant versus cells not treated with resveratrol; two-way ANOVA). The half-lifes of the respective mRNAs are IL-8: CM 20.39 ± 3.06 min, CM + Res 11.02 ± 1.60 min; TNF-α: CM 19.63 ± 6.10 min, CM + Res 7.87 ± 1.29 min.

To verify the inhibitory post-transcriptional effect on pro-inflammatory gene expression in a second human cell type, being more relevant for immune reactions, we measured the effects of resveratrol on pro-inflammatory gene expression in human monocytic Mono Mac 6 cells. As induction of iNOS expression in human monocyte/macrophages cell lines seems to be impossible (34), we concentrated on IL-8 and TNF-α mRNA expression and stability. As shown in Figure 3, resveratrol markedly inhibited cytokine-induced TNF-α or IL-8 mRNA expression (Figure 3A and B). Similar to DLD-1 cells, DRB-decay experiments demonstrated that this effect is related to the resveratrol-mediated destabilization of the mRNAs (Figure 3C and D).

### Protein-fishing experiments reveal KSRP as a direct binding partner and target of resveratrol

Several effects of resveratrol were attributed to its ability to activate the histone deacetylase SIRT1 (12). However, pharmacological (by sirtinol) or molecular (by siRNA) inhibition of SIRT1 (see Supplementary Figure S3) did not influence the effect of resveratrol on human iNOS mRNA expression, indicating a SIRT1-independent mechanism.

**Figure 4.**
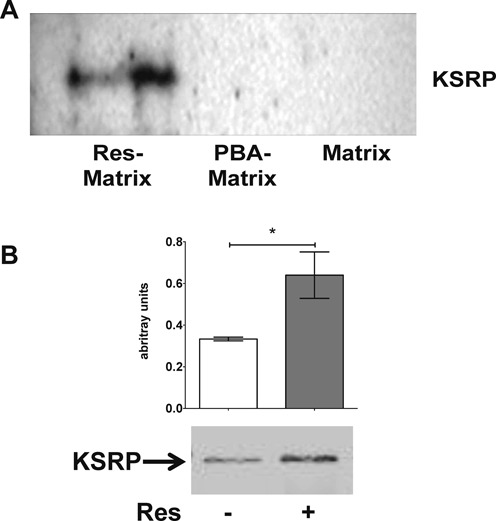
Resveratrol specifically binds to KSRP. (A) Western blot analyses of KSRP in eluates from resveratrol-, PBA- and matrix-fished proteins from PBMC lysates. Data shown are representative of three independent experiments. (B) DARTS of KSRP in the presence of vehicle (0.7% DMSO) or 30 μM resveratrol. DARTS is a method to validate ligand-target interaction by the reduction in the protease susceptibility of the target protein (here pronase was used as protease and KSRP as target) due to binding of the ligand (here resveratrol). The picture (lower panel) shown is representative of three independent experiments. Densitometry analysis (upper panel) shows the integrated intensities of respective bands (*P < 0.05 versus 0.7% DMSO; *t*-test).

Therefore, we started to evaluate the molecular mechanism of the resveratrol-mediated mRNA decay. At first, we performed target-fishing experiments to identify direct resveratrol binding partners, which could be involved in post-transcriptional regulation processes. For bead-immobilization we connected a linker molecule to the 4′-OH-group of resveratrol (Supplementary Figure S4A), whose etherification has been shown not to hinder its biological activity (11). Phenoxybutyric acid (PBA, mimicking the resveratrol structure) was used as a negative control (Supplementary Figure S4B). In protein-fishing experiments these beads were incubated with human PBMC lysates. Comparison of resveratrol- and PBA-fished proteins (Supplementary Figure S4C) showed a higher presence of proteins fished by resveratrol for spot number 2, 5 and 6. All three spots were identified as human KSRP by mass spectrometry. The selective binding of resveratrol to KSRP was confirmed by western blot (Figure 4A). These experiments identified the RNA binding protein KSRP as a selective high affinity-binding partner of resveratrol.

### Resveratrol stabilizes human KSRP protein

To confirm the direct binding of resveratrol with KSRP, we used a drug affinity responsive target stability (DARTS) approach (35), which validates ligand-target interaction by the reduction of the protease susceptibility of the protein target (here KSRP) due to binding of the ligand (resveratrol). Thus, isolated KSRP was incubated with resveratrol and subjected to treatment with the protease pronase. As shown in Figure 4B, resveratrol protected KSRP from proteolytic degradation, supporting the direct interaction of resveratrol with KSRP.

### Downregulation of KSRP expression inhibits the destabilizing effect of resveratrol on IL-8, iNOS or TNF-α mRNA

**Figure 5.**
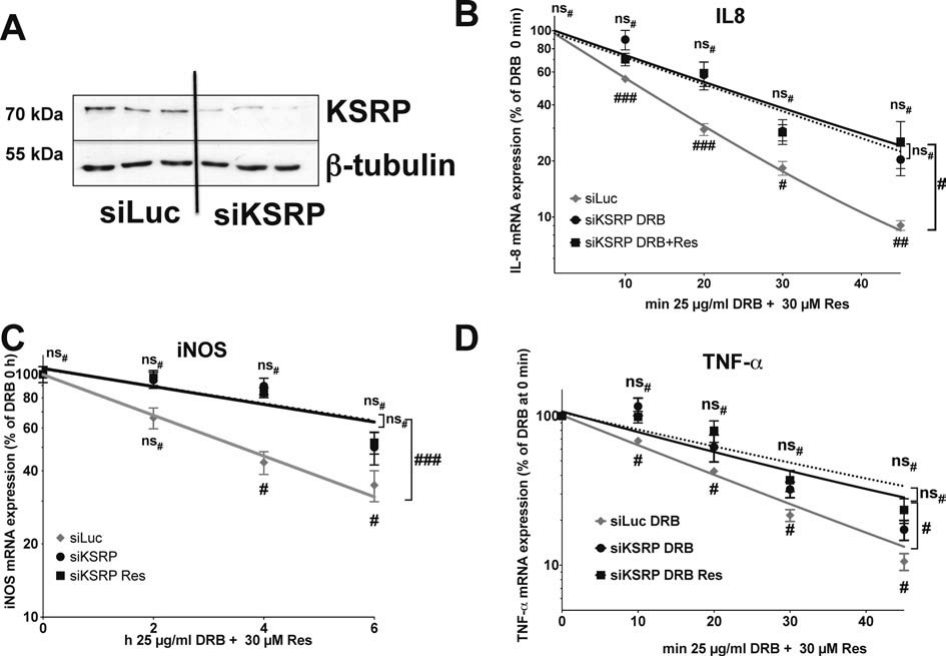
Resveratrol reduces the stability of IL-8, iNOS and TNF-α mRNA in human DLD-1 cells in a KSRP-dependent manner. DLD-1 cells were stably transfected with constructs resulting in the intracellular expression of KSRP siRNA (siKSRP) or Luc siRNA (siLuc, control). (A) Expression of KSRP (KSRP) in siKSRP and siLuc cells was analyzed by western blot experiments using a specific anti-KSRP antibody. For normalization, the expression of β-tubulin (β-tubulin) was analyzed in parallel using a specific anti-β-tubulin antibody. Shown is one of four representative western blots. (B–D) DLD1 siLuc or siKSRP cells were treated with CM for 2 h (B and D) or 4 h (C). Note that 30 μM resveratrol was added and 30 min later the cells were treated with DRB (25 μg/ml). After 10, 20, 30, 45 min (B and D) or 2, 4 and 6 h (C) RNA was isolated and the expression of IL-8, iNOS, TNF-α and GAPDH (for normalization) mRNA was measured by qRT-PCR. The IL-8, iNOS or TNF-α mRNA expression was normalized to the GAPDH mRNA expression. (B) The relative IL-8 mRNA expression after 2 h CM was set to 100%. Shown are the mean ± SEM of *n* = 8–10 analyses (###*P* < 0.001; ##*P* < 0.01; #*P* < 0.05; ns_#_, not significant versus siKSRP cells not treated with resveratrol, two-way ANOVA). The half-lifes of the IL-8 mRNAs under the different conditions are: siLuc CM 10.09 ± 0.70 min; siKSRP: CM 19.76 ± 4.18 min, siKSRP CM + Res 20.88 ± 3.29 min. (C) The relative iNOS mRNA expression after 4 h CM was set to 100%. Shown are the mean ± SEM of *n* = 10–12 analyses (###*P* < 0.001; #*P* < 0.05; ns_#_, not significant versus siKSRP cells not treated with resveratrol; two-way ANOVA). The half-lifes of the iNOS mRNAs under the different conditions are: siLuc CM 4.31 ± 0.75 h; siKSRP: CM 7.60 ± 2.88 h, siKSRP CM + Res 7.83 ± 2.36 h. (D) The relative TNF-α mRNA expression after 2 h CM was set to 100%. Shown are the mean ± SEM of *n* = 12–14 analyses (#*P* < 0.05; ns_#_, not significant versus siKSRP cells not treated with resveratrol; two-way ANOVA). The half-lifes of the TNF-α mRNAs under the different conditions are: siLuc CM 14.85 ± 0.94 min; siKSRP: CM 20.17 ± 4.29 min, siKSRP CM + Res 24.81 ± 4.37 min.

As KSRP is an important regulator of multiple inherently instable mRNAs mostly coding for pro-inflammatory mediators, the data above imply that resveratrol destabilizes the iNOS, IL-8 or TNF-α mRNA by modulating the mRNA binding activity of KSRP. To analyze the effect of resveratrol on pro-inflammatory gene expression in the absence of KSRP, we generated DLD-1 cells with a diminished KSRP-expression (**siKSRP**; see Figure 5A). As control we generated DLD-1 cells with a stable expression of an anti-luciferase siRNA (**siLuc**). In accordance to published data, siRNA-mediated downregulation of KSRP expression enhanced iNOS, IL-8 and TNF-α mRNA expression (Supplementary Figure S5A–C). More importantly, resveratrol (30 μM) reduced iNOS, IL-8 and TNF-α mRNA expression only in cells with normal but not in cells with diminished KSRP expression (Supplementary Figure S5D–F). This hints toward an important role of KSRP in post-transcriptional resveratrol effects.

Furthermore, we measured the effect of resveratrol on IL-8, iNOS and TNF-α mRNA stability in siKSRP cells. As control the mRNA decay in DLD-1 cells with normal KSRP expression (siLuc) was determined in parallel. We pre-incubated cells with CM and resveratrol, added DRB and residual IL-8, iNOS or TNF-α mRNA amounts were measured by qRT-PCR at the time points indicated. In CM-treated DLD-1 siKSRP cells the stability of IL-8, iNOS or TNF-α mRNA was significantly enhanced in comparison to DLD-1 siLuc cells (see Figure 5B–D). However, most importantly, resveratrol treatment had no effect on IL-8, iNOS or TNF-α mRNA stability in DLD-1 siKSRP cells. These data indicate that resveratrol reduces IL-8, iNOS or TNF-α expression by KSRP-dependent destabilization of the mRNAs.

### Inactivation of the KSRP gene enhances CXCL-1, iNOS and TNF-α expression and blocks the inhibitory effect of resveratrol on pro-inflammatory gene expression

**Figure 6.**
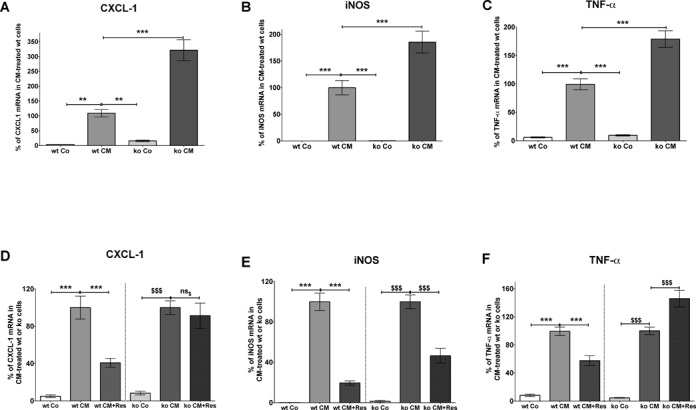
Inactivation of the KSRP gene enhances CXCL-1, iNOS and TNF-α mRNA expression and alleviates the resveratrol effect in murine peritoneal cells. Peritoneal cells were isolated from KSRP^+/+^ (wt) and KSRP^−/−^ (ko) mice. Adherent cells (mostly monocytes/macrophages) were used for the experiments. (A–C) Adherent peritoneal cells were incubated with LPS (2 μg/ml) and IFN-γ (100 U/ml) to induce pro-inflammatory mRNA expression. After 2–6 h cells were lyzed and the expression of CXCL-1, iNOS, TNF-α and GAPDH (for normalization) mRNA was measured. CXCL-1, iNOS and TNF-α mRNA expression was normalized to GAPDH mRNA expression. The CM-induced mRNA expression in KSRP^+/+^ (wt) cells was set to 100%. Shown are the mean ± SEM of *n* = 8–10 analyses (***P < 0.001; **P < 0.01 versus CM-treated cells isolated from KSRP^+/+^ mice; one-way ANOVA). (D–F) Adherent peritoneal cells were pre-incubated with 30 μM resveratrol (Res) for 1 h and then treated with LPS (2 μg/ml) and IFN-γ (100 U/ml) for additional 2–6 h. RNA was isolated and the expression of CXCL-1, iNOS, TNF-α and GAPDH (for normalization) mRNA was measured by qRT-PCR. CXCL-1, iNOS and TNF-α mRNA expression was normalized to GAPDH mRNA expression. The mRNA expression in CM-treated KSRP^+/+^ (wt) or KSRP^−/−^ cells (ko) was set to 100%. Shown are the mean ± SEM of *n* = 8–10 analyses (***P < 0.001 versus CM-treated cells isolated from KSRP^+/+^ mice; $$$*P* < 0.001; ns_$_, not significant versus CM-treated cells isolated from KSRP^−/−^ mice; one-way ANOVA).

We analyzed the effect of KSRP-deficiency on CXCL-1 (the murine IL-8 functional homolog (36)), iNOS and TNF-α mRNA expression in adherent primary peritoneal cells isolated from KSRP^+/+^ and KSRP^−/−^ mice. The cells were pre-incubated with or without 30 μM resveratrol for 1 h and pro-inflammatory gene expression was induced by incubation with LPS (2 μg/ml) and IFN-γ (100 U/ml) for 2 or 6 h. As shown in Figure 6A–C, inactivation of the KSRP gene resulted in markedly enhanced CXCL-1, iNOS and TNF-α mRNA expression. Furthermore, the inhibitory effect of resveratrol on CXCL-1, iNOS or TNF-α mRNA expression was reduced or totally blocked in KSRP^−/−^ cells (see Figure 6D–F).

### Resveratrol prevents p38 MAPK-dependent threonine-phosphorylation of KSRP without changing p38 MAPK activation or activity

**Figure 7.**
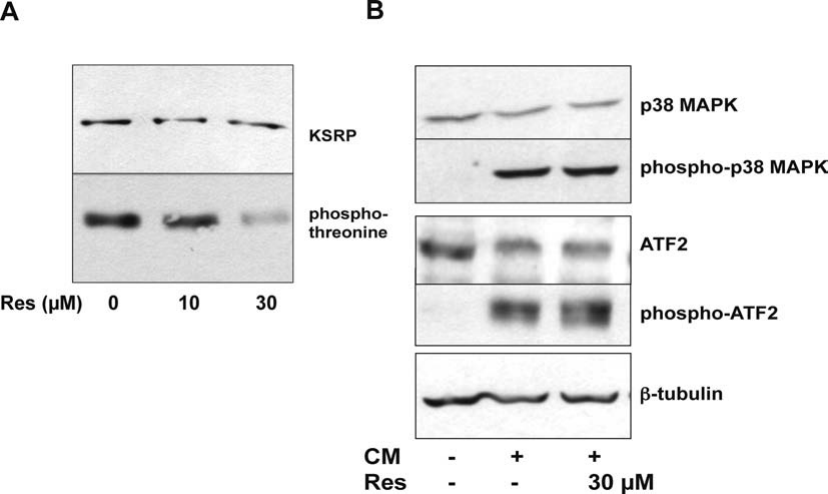
Resveratrol reduces the p38 MAPK-dependent KSRP phosphorylation. (A) DLD-1 cells were pre-treated for 1 h with 10 or 30 μM resveratrol and incubated with CM for 6 h. Cell lysates were prepared and subjected to immunoprecipitation using a polyclonal anti-KSRP antibody. The immunoprecipated material was separated by SDS-PAGE and the amounts of total KSRP protein (KSRP) and of threonine-phosphorylated KSRP (phospho-threonine) were analyzed by western blot experiments using specific antibodies. One representative out of four co-immunoprecipitation assays is shown. (B) DLD-1 cells were pre-treated with 30 μM resveratrol and incubated with CM for 15 min. Then, protein extracts were prepared. The extracts were analyzed for p38 MAPK, phospho-p38 MAPK, ATF2, phospho-ATF2 and β-tubulin (for normalization) expression by western blot using the specific antibodies described. One representative out of four western blot analyses is shown.

As shown in Supplementary Figure S6, resveratrol did not change KSRP protein expression. Therefore, it seems very likely that resveratrol may influence p38 MAPK-, ATM-kinase- or PI3K-Akt-dependent KSRP protein phosphorylation, which has been shown to modify KSRP activity (27). Only inhibition of p38 MAPK reduced cytokine-induced pro-inflammatory gene expression (see Supplementary Figure S7 and (29)). Thus, we analyzed the resveratrol effect on p38 MAPK-dependent phosphorylation of KSRP in more detail. Briata *et al.* showed that phosphorylation of KSRP at threonine 692 by p38 MAPK inactivates KSRP (37). To analyze if resveratrol binding to KSRP interferes with this phosphorylation, KSRP was immunoprecipitated from extracts of CM-treated DLD-1 cells incubated with or without resveratrol. The amount of KSRP and its phosphorylation at threonine residues was determined using specific antibodies. As shown in Figure 7A, while immunoprecipitating equal amounts of KSRP in all samples, phosphorylation of threonine residues was significantly reduced in resveratrol-treated cells. Therefore, it is likely that direct binding of resveratrol to KSRP enhances KSRP activity by reducing the inhibitory phosphorylation at threonine 692.

p38 MAPK is a major post-transcriptional regulator of gene expression (38) and is crucially involved in human iNOS, IL-8 and TNF-α expression (29,39–40). Activation of p38 MAPK is mediated by phosphorylation by MAPK kinases (41). We examined whether resveratrol blocks KSRP phosphorylation by interfering with p38 MAPK activation by analyzing the effect of resveratrol on CM-induced p38 MAPK phosphorylation in DLD-1 cells. As shown in Figure 7B, CM-incubation markedly enhanced p38 MAPK phosphorylation. This effect was not blocked by resveratrol, indicating that resveratrol does not interfere with p38 MAPK activation. To assess the effect of resveratrol on p38 MAPK activity, we analyzed ATF2 phosphorylation in DLD-1 cells, as ATF2 is a direct substrate of p38 MAPK (42). As shown in Figure 7B, incubation of DLD-1 cells with resveratrol did not inhibit cytokine-induced ATF2 phosphorylation. Therefore, in DLD-1 cells resveratrol neither inhibits p38 MAPK activation nor activity.

### Mutation of threonine 692 to alanine in KSRP abolishes the inhibitory effect of resveratrol on pro-inflammatory gene expression

**Figure 8.**
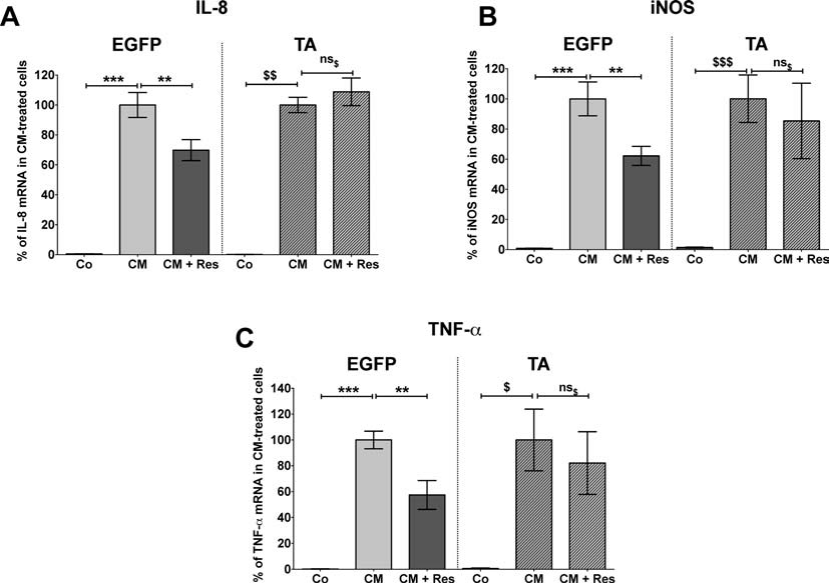
Mutation of threonine 692 to alanine blocks resveratrol-mediated reduction of pro-inflammatory gene expression. DLD-1 cells were transduced with lentiviral constructs resulting in the expression of EGFP (control) or EGFP-KSRP(T692A) fusion protein (TA). EGFP or TA cells were pre-treated with or without 30 μM resveratrol and then incubated with CM for 2 h (IL-8 and TNF-α) or 6 h (iNOS). RNA was isolated and the expression of iNOS, IL-8, TNF-α and GAPDH (for normalization) mRNA was measured by qRT-PCR. The iNOS, IL-8 or TNF-α mRNA expression was normalized to the GAPDH mRNA expression. The relative IL-8 (A), iNOS (B) and TNF-α (C) mRNA expression in CM-treated EGFP (left panel) or TA (right panel) cells was set to 100%. Shown are the mean ± SEM of *n* = 4–6 analyses (***P < 0.001; **P < 0.01 versus CM-treated EGFP cells; $$$*P* < 0.001; $$*P* < 0.01; $*P* < 0.05; ns_$_, not significant versus CM-treated TA cells; one-way ANOVA).

Analysis of the KSRP amino acid sequence showed several conserved MAPK phosphorylation sites (SP/TP) and mutation of threonine 692 to alanine in one of these MAPK sites inhibits the inactivating p38 MAPK phosphorylation of KSRP (37). To analyze whether mutation of threonine 692 to alanine modifies the effect of resveratrol on pro-inflammatory gene expression, we created lentiviral transduced DLD-1 cells expressing an EGFP-KSRP(T692A) fusion protein (**TA** cells) or expressing **EGFP** (as control). We confirmed expression of the EGFP-KSRP(T692A) fusion protein in western blot analyses (see Supplementary Figure S8). As shown in Figure 8A–C, overexpression of EGFP-KSRP(T692A) in DLD-1 cells reduced or abolished the inhibitory effect of resveratrol on CM-induced IL-8, iNOS and TNF-α mRNA expression.

### Resveratrol enhances the interaction of KSRP with the exosome

KSRP-mediated ARE-dependent mRNA decay has been described to depend on the interaction of KSRP with the exosome (23,43). To test if resveratrol influences the KSRP-exosome interaction, KSRP was immunoprecipitated from extracts of CM-treated DLD-1 cells incubated with or without resveratrol. The amount of KSRP and the exosomal component PM-Scl in the precipitates was determined using specific antibodies. As shown in Supplementary Figure S9A, equal amounts of KSRP were immunoprecipitated from all samples. Resveratrol incubation resulted in enhanced amounts of the exosomal component PM-Scl in the precipitates indicating a stimulation of the KSRP-exosome interaction by resveratrol (see Supplementary Figure S9B).

### Resveratrol enhances the intra-cellular interaction of KSRP with the IL-8, iNOS and TNF-α mRNA

**Figure 9.**
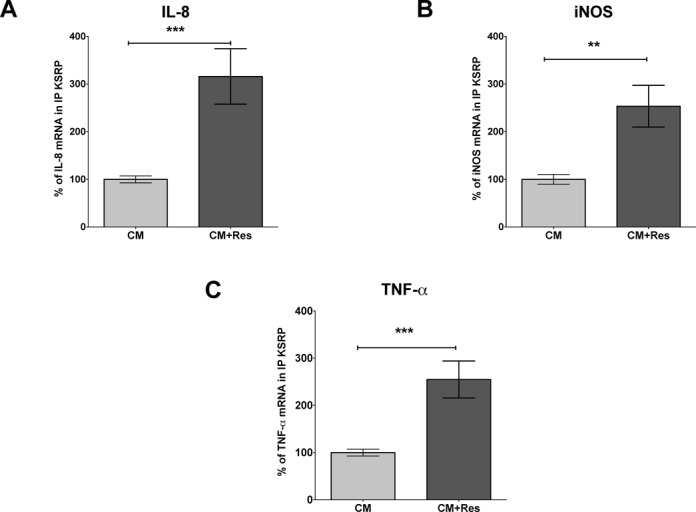
Resveratrol incubation enhances intracellular binding of KSRP to the IL-8, iNOS and TNF-α mRNA. DLD-1 cells were incubated for 2 (A and C) or 4 h (B) with CM with or without 30 μM resveratrol (Res). Cells were lyzed and RNA bound by KSRP protein was immunoprecipitated by a specific antibody. Immunoprecipitation with immunoglobulin G (IgG) was used as negative control. To normalize for subsequent RNA purification steps, 1 ng/sample *in vitro* transcribed luciferase RNA was added before the RNA was isolated from immunoprecipitated proteins. The amounts of IL-8, iNOS and TNF-α mRNA bound by KSRP were determined by qRT-PCR using the luciferase RNA as normalization control. (A) A summary of 14 immunoprecipitation-qRT-PCR analyses is shown. Columns (means ± SEM) represent relative IL-8 mRNA levels bound by KSRP (***P < 0.001 versus CM-treated DLD-1 cells; *t*-test). (B) A summary of 12 immunoprecipitation-qRT-PCR analyses is shown. Columns (means ± SEM) represent relative iNOS mRNA levels bound by KSRP (**P < 0.01 versus CM-treated DLD-1 cells, *t*-test). (C) A summary of 16 immunoprecipitation-qRT-PCR analyses is shown. Columns (means ± SEM) represent relative TNF-α mRNA levels bound by KSRP (***P < 0.001 versus CM-treated DLD-1 cells; *t*-test).

To test if resveratrol is able to increase KSRP-RNA binding activity in intact cells, we analyzed intra-cellular binding of KSRP to the IL-8, iNOS and TNF-α mRNA by immunoprecipitation-qRT-PCR assays (see Figure 9). These analyses showed resveratrol-dependent enhancement of KSRP binding to these mRNAs. These results may explain the resveratrol-mediated destabilization of pro-inflammatory mRNAs and therefore the anti-inflammatory effect of the substance.

## DISCUSSION

In this study we show that resveratrol post-transcriptionally dampened pro-inflammatory gene expression in human and murine cells. By target-fishing experiments, we identified KSRP as a direct intracellular binding protein of resveratrol and verified the importance of KSRP for the post-transcriptional effects of resveratrol. In addition, we demonstrate that resveratrol prevents the p38 MAPK-dependent inhibitory threonine phosphorylation of KSRP and thereby enhances KSRP binding to target mRNAs, as well as the interaction with the exosome. This seems to be the molecular mechanism explaining the anti-inflammatory effects of resveratrol.

KSRP is a multifunctional protein involved in c-myc promoter activation, c-src hnRNA splicing, apoB mRNA editing, neuronal mRNA localization, thrombin mRNA 3′-end processing, ARE-mediated mRNA decay and regulation of translation, as well as miRNA maturation (26,44–45). In the present study we analyzed the impact of resveratrol on ARE-mediated mRNA decay, to elucidate the biological role of the direct resveratrol-KSRP interaction. We focused our analysis on CXCL-1/IL-8, iNOS and TNF-α expression for two reasons. First, it is known that the expression of these pro-inflammatory genes can be regulated by resveratrol (9,46–47) and, second, the decay of all these mRNAs depends on the interaction of KSRP with AREs in their 3′-UTR sequences (25,43,48).

In human epithelial DLD-1 and monocytic Mono Mac 6 cells resveratrol decreased the expression of all analyzed KSRP-target genes (see Figure 1). Also in primary peritoneal cells isolated from KSRP wild-type mice resveratrol reduced LPS/IFN-γ-induced CXCL-1 (the murine functional IL-8-homolog), iNOS and TNF-α mRNA expression (see Figure 6D–F). In accordance, reduced expression of these key inflammatory mediators after resveratrol-treatment has been described also in various other cell types (47,49) from different species (human, mouse, rat) or in tissues from several animal disease models (50,51). This inhibition was primarily attributed to resveratrol-mediated inhibition of NF-κB activation. However, also enhancement of iNOS or TNF-α expression by resveratrol (52) or no effect on iNOS, IL-8 or TNF-α expression (53–55) have been reported.

The expression of CXCL-1/IL-8, iNOS and TNF-α is regulated by transcriptional and post-transcriptional mechanisms (15,56–58). In reporter gene analyses (see Supplementary Figure S1), resveratrol unexpectedly *enhanced* cytokine-induced human iNOS and IL-8 promoter activity in DLD-1 cells and inhibited TNF-α promoter activity only at 30 μM. The resveratrol-related enhancement of the human iNOS and IL-8 promoter activity might be explained by the resveratrol-dependent increase of NF-κB and/or STAT-1α activity (see Supplementary Figure S1D and E). As shown in rat mesangial cells (59), A7r5 rat aortic smooth muscle cells (54) and murine RAW macrophages (53), the reduced iNOS, IL-8 or TNF-α expression in human DLD-1 cells after resveratrol treatment cannot be explained only by impaired promoter activity. In contrast, Chung *et al.* and Tsai *et al.* described reduced iNOS expression and promoter activity in murine RAW 264.7 macrophages after resveratrol treatment (9). As several data imply different regulation of murine, rat and human iNOS promoter activity (56), some of these opposing data may be explained by species differences. In summary, the decreased expression of iNOS, IL-8 and TNF-α could not be explained by resveratrol-mediated effects on the promoter activity.

Our data show that resveratrol mediates its effect on iNOS, IL-8 and TNF-α expression by decreasing the stability of the mRNA (see Figures 2 and 3). The resveratrol effect on pro-inflammatory gene expression is essentially dependent on KSRP, since it is abolished by reduced KSRP expression (siKSRP cells, see Figure 5 and Supplementary Figure S5). KSRP mediates the decay of several pro-inflammatory mRNAs by binding to AREs in the 3′-UTR and recruiting 5′- and 3′-decay enzymes (26). The general importance of KSRP in the regulation of mRNA decay is demonstrated by the increase of the half-life of iNOS, IL-8 and TNF-α mRNA in siKSRP cells (see Figure 5 and (25)). These results emphasize the importance of the interaction of resveratrol with KSRP for its effect on the decay of human pro-inflammatory mRNAs.

Also in primary peritoneal cells isolated from mice, inactivation of the KSRP gene resulted in enhanced expression of CXCL-1, iNOS and TNF-α mRNA, indicating a major effect of KSRP on the expression of these pro-inflammatory genes in the murine system (see Figure 6A–C). Since the inhibitory effect of resveratrol of pro-inflammatory gene expression is reduced (iNOS) or abolished (CXCL-1 and TNF-α) in peritoneal cells of KSRP^−/−^ mice (see Figure 6D–F), these results support our hypothesis that major anti-inflammatory effects of resveratrol are KSRP-dependent.

Several effects of resveratrol on gene expression are attributed to the SIRT1-mediated inhibition of the activity of transcription factors like NF-κB (12). In contrast, in the current study we show evidence that the inhibitory effect of resveratrol on CM-induced iNOS mRNA expression (see Supplementary Figure S3) is SIRT1-independent. Regulation of pro-inflammatory gene expression (e.g. human IL-8, iNOS and TNF-α expression) strongly depends on post-transcriptional mechanisms (60). The fact, the observed anti-inflammatory resveratrol effects in DLD-1 cells being independent of SIRT1 provides another evidence that resveratrol rather modifies post-transcriptional mechanisms instead of interfering with the promoter activity.

In a previous study we have demonstrated the important role of the 3′-UTR in the complex regulation of human iNOS expression by different RNA binding proteins (25). Accordingly, resveratrol reduced luciferase expression only in cells transfected with constructs containing the human iNOS-3′-UTR (see Supplementary Figure S2). This supports the idea that resveratrol enhances the RNA binding activity of KSRP to 3′-UTR sequences and thereby promotes the decay of the corresponding mRNAs. Comparable to human iNOS 3′-UTR, also the IL-8 and TNF-α 3′-UTR contain several AREs, which control the stability of these mRNAs and serve as binding sites for RNA-binding proteins like KSRP and HuR (24,61). For this reason it is very likely that the resveratrol effect on IL-8 and TNF-α mRNA decay is mediated via KSRP binding to these sequence elements in the 3′-UTR. In the literature only few examples indicate post-transcriptional effects of resveratrol. For example, resveratrol stabilizes eNOS mRNA in human EA.hy 926 endothelial cells (16). In human Jurkat T cells, transfection experiments using constructs containing the 3′-UTR of the TNF-α mRNA demonstrated that resveratrol post-transcriptionally suppressed TNF-α expression. This effect was blocked by overexpression of the RNA binding protein, HuR, known to stabilize ARE-containing mRNAs (18). Inverse regulation of several KSRP mRNA targets by binding of HuR to the same or similar 3′-UTR sequences has been published for iNOS (25), IL-8 (24) and TNF-α (61). Therefore, it is tempting to speculate that the resveratrol induced changes in the mRNA stability of HuR-targeted transcripts (as shown in Jurkat T cells) are related to an enhanced 3′-UTR binding of KSRP and replacement of HuR from the 3′-UTR sequences.

The resveratrol-mediated effects on KSRP seem to depend on post-translational mechanisms since neither cytokine incubation nor resveratrol treatment influenced KSRP protein expression in DLD-1 cells (Supplementary Figure S7).

Protein phosphorylation of KSRP by different kinases has been described to regulate KSRP activity and localization (27). p38 MAPK-mediated phosphorylation of KSRP at threonine 692 lowers its affinity for AREs, thus, stabilizing transcripts whose stability is regulated by KSRP (37). Phosphorylation of KSRP by PI3K-AKT at S193 promotes the unfolding of the KH1 domain of KSRP resulting in enhanced binding of 14-3-3ζ and relocation of KSRP to the nucleus, which prevents its role in cytoplasmic mRNA decay (62). In addition, S193 phosphorylation decreases the ability of KSRP to associate with the exosome and inhibits mRNA decay (37). ATM is another protein kinase shown to phosphorylate KSRP (63). ATM-mediated phosphorylation results in enhanced KSRP-regulated maturation of miRNAs. Treatment with specific inhibitors of these kinases (see Supplementary Figure S8) indicated that phosphorylation by p38 MAPK seems to be the most important KSRP-related mechanism regulating pro-inflammatory gene expression in DLD-1 cells. p38 MAPK phosphorylates KSRP at threonine 692. This phosphorylation inhibits binding of KSRP to several mRNAs thereby enhancing their stability. In fact, in DLD-1 cells resveratrol treatment reduced phosphorylation of threonine residues in KSRP (Figure 7A). Resveratrol has been described to inhibit (64), to enhance (65) or to have no effect (66) on p38 MAPK activation in different cell types. However, as shown in Figure 7B, phosphorylation of ATF2, another p38 MAPK substrate, was not reduced. This suggests that the direct binding of resveratrol to KSRP may prevent p38 MAPK-mediated threonine 692 phosphorylation, likely by steric hindrance. As phosphorylation at threonine 692 inhibits KSRP-RNA binding activity, the resveratrol-mediated blockage of this threonine-phosphorylation of KSRP is likely the explanation for the stimulatory effect of this compound on KSRP activity detected in our study. Supporting this hypothesis, overexpression of a non-phosphorylatable mutant of KSRP (EGFP-KSRP(T692A)) in DLD-1 cells abolished the resveratrol effect on pro-inflammatory gene expression (see Figure 8D–F).

KSRP-mediated ARE-dependent mRNA decay has been described to depend on the interaction of KSRP with the exosome (23,43). Therefore, we analyzed the effect of resveratrol on the interaction of KSRP with the exosome. As seen in Supplementary Figure S9, resveratrol enhanced the KSRP-exosome interaction indicating enhanced mRNA degradation after resveratrol treatment.

As KSRP-mediated mRNA decay depends on the interaction of KSRP with mRNAs, we finally tested the effects of resveratrol on the intra-cellular binding of KSRP to the IL-8, iNOS or TNF-α mRNA by IP-qRT-PCR. A shown in Figure 9, resveratrol enhanced the KSRP-mRNA interaction, also indicating enhanced mRNA degradation after resveratrol treatment.

In summary, our data obtained in human DLD-1 and Mono Mac 6 cells, as well as in primary peritoneal cells of KSRP^+/+^ and KSRP^−/−^ mice, show that resveratrol specifically binds to KSRP and thereby prevents (likely by steric hindrance) the inhibitory phosphorylation at threonine 692 of this important RNA binding protein. The lack of threonine 692 phosphorylation results in enhanced binding of KSRP to its target mRNAs and enhanced KSRP-exosome interaction, resulting in augmented ARE-mediated KSRP-dependent mRNA decay. Thus, resveratrol regulates pro-inflammatory gene expression on the post-transcriptional level by increasing the KSRP-mediated mRNA decay.

KSRP is one of the few direct resveratrol interacting proteins yet identified. As KSRP has been shown to be involved in diverse transcriptional and/or post-transcriptional mechanisms, KSRP may be a key mediator explaining the pleiotropic effects of resveratrol.

## SUPPLEMENTARY DATA

Supplementary Data are available at NAR Online.

SUPPLEMENTARY DATA
