# Supplementary material for: Resveratrol post-transcriptionally regulates pro-inflammatory gene expression via regulation of KSRP RNA binding activity
Source: Nucleic Acids Res. 2014 Oct 28;42(20):12555–69. doi: 10.1093/nar/gku1033 (PMC4227754; doi:10.1093/nar/gku1033)
Supplement: SUPPLEMENTARY DATA [file supp_42_20_12555__index.html]

Resveratrol post-transcriptionally regulates pro-inflammatory gene expression via regulation of KSRP RNA binding activity — Resveratrol post-transcriptionally regulates pro-inflammatory gene expression via regulation of KSRP RNA binding activity — SUPPLEMENTARY DATA 

# Resveratrol post-transcriptionally regulates pro-inflammatory gene expression via regulation of KSRP RNA binding activity

## SUPPLEMENTARY DATA

**Files in this Data Supplement:**

- SUPPLEMENTARY DATA
