# Supplementary material for: Resveratrol post-transcriptionally regulates pro-inflammatory gene expression via regulation of KSRP RNA binding activity
Source: Nucleic Acids Res. 2014 Oct 28;42(20):12555–69. doi: 10.1093/nar/gku1033 (PMC4227754; doi:10.1093/nar/gku1033)
Supplement: SUPPLEMENTARY DATA [file supp_gku1033_nar-00840-x-2014-File011.pdf]

SUPPLEMENTARY DATA

SUPPLEMENTARY MATERIALS AND METHODS

Materials – Trypsin, glutamine, pyruvate-solutions, PMSF, dithiothreitol, leupeptin, aprotinin, agarose, LPS, DRB, BSA, resveratrol, sirtinol, chloroquine sulfate, protamine sulfate, polybrene, P8340 protease inhibitor cocktail, the anti-SIRT1 siRNA (SASI-Hs01-00153666, 5-GUGUCAUGGUUCCUUUG-CA[dT][dT]), all oligonucleotides and dual labeled probes used, horseradish-peroxidase-coupled anti-mouse IgG, the monoclonal anti-β-tubulin, anti-GAPDH, and anti-phospho threonine antibodies were purchased from Sigma, Deisenhofen, Germany. RNasin, Pronase, complete EDTA-free protease and phosphatase inhibitor cocktail were obtained from Roche Diagnostics, Mannheim, Germany. All cell culture grade plastic material was obtained from Greiner, Solingen, Germany. Toyopearl® AF 650 Amino beads were obtained from Tosoh Bioscience, King of Prussia, USA. CyDyes (Cy3 and Cy5) and the Hybond-C extra nitrocellulose membrane were obtained from GE Healthcare, Freiburg, Germany. The peqGOLD IV protein marker was obtained from PeqLab, Erlangen, Germany. The enhanced chemiluminescence detection system (ECL) was obtained by PerkinElmer, Rodgau, Germany. The p38 MAPK inhibitor 4-(4-Fluorophenyl)-2-(4-methylsulfinylphenyl)-5-(4-pyridyl)1H-imidazole (SB 203580), the ATM kinase inhibitor 2-Morpholin-4-yl-6-thianthren-1-yl-pyran-4-one (KU55933), and the phosphatidylinositol 3-kinase (PI3K-AKT) inhibitor wortmannin were from Calbiochem/Merck, Darmstadt, Germany. The GeneJuice transfection reagent was from Merck, Darmstadt, Germany. The AllStars Neg. Control siRNA (1027281) and the HiPerFect HTS Reagent were from Qiagen, Hilden, Germany. Restriction enzymes, Taq DNA polymerase, Klenow DNA polymerase, dNTPs, NTPs, the polyclonal antibodies against p38 MAPK, phospho-p38 MAPK, and phospho-ATF2 were purchased from New England Biolabs, Frankfurt a.M., Germany. The polyclonal anti-ATF2 antibody and A/G agarose beads were obtained from Santa Cruz Biotechnology, Heidelberg, Germany. The polyclonal anti-KSRP antibody was obtained from Novus Biologicals, Cambridge, UK. The polyclonal anti-PM-Scl 100 antibody was a kind gift of Dr. G. Pruijn (Department of Biochemistry, University of Nijmegen, The Netherlands). The High-Capacity cDNA Reverse Transcription Kit, was purchased from Applied Biosystems, Darmstadt, Germany. The ABsolute™ QPCR SYBR Green Fluorescein Mix Kit was obtained from Thermo-Fisher Scientific, Surry, UK. The Dual-Luciferase-Reporter-Assay-System was obtained from Promega, Heidelberg, Germany. Human and murine interferon-γ (IFN-γ), interleukin-1β (IL-1β), and tumor necrosis factor-α (TNF-α) were obtained from Milteny Biotech, Bergisch Gladbach, Germany. FCS and DMEM were purchased from PAN-Systems, Nürnberg, Germany. Zeocin and psiRNAhH1-GFPzeo were purchased from InvivoGen, San Diego, USA. pcDNA4/TO and pEGFP-C1 were purchased from Invitrogen, Groningen, The Netherlands. The pathway detection plasmids pAP1-Luc, pNF-κB-Luc, and pGAS-Luc were obtained from Stratagene, Heidelberg, Germany. pMIR-SHIP1 (1) was obtained from Addgene, Cambridge, USA. The HIV-1-derived lentiviral vectors pCDH-CMV-MCS-EF1-copGFP and pCDH-CMV-MCS-EF1-Puro were obtained from SBI, Mountain View, CA, USA. The pGIPZ Lentiviral shRNA expression vector (pGIPZ-hmr-siKSRP) coding for microRNA-adapted shRNA directed against human, murine, and rat KSRP was obtained from Thermo-Fisher Scientific, Schwerte, Germany. The Bradford reagent

mix and Ready Strip™ immobilized pH gradient (IPG) strips were obtained from Bio-Rad Laboratories, Munich, Germany. The monoclonal anti-KSRP antibody was a kind gift of Dr. Douglas L. Black, Howard Hughes Medical Institute at UCLA, Los Angeles, USA. The plasmid pGL3-TNF-α-prom containing the human TNF-α promoter in front of the luciferase reportergene (2) was a kind gift of Dr. G. Erkel, Department of Molecular Biotechnology and Systems Biology, Kaiserslautern, Germany. The plasmid pUHC13-IL8-prom containing the human IL-8 promoter in front of a luciferase reporter gene (3) was a kind gift of Dr. M. Kracht, Rudolf-Buchheim-Institute of Pharmacology, Justus-Liebig-University Giessen, Giessen, Germany. The plasmid pCMV-TAG2B-KSRP(T692A) (4) containing a mutated KSRP (threonine to alanine at position 692) was a generous gift of R. Gherzi, Gene Expression Regulation Laboratory, IRCCS Azienda Ospedaliera Universitaria San Martino-IST, Genova, Italy.

Sequences of the oligonucleotides used as sense and antisense primers as well as Taqman hybridization probes in qRT-PCR experiments

human iNOS	
sense	5’-TGCAGACACGTGCGTTACTCC-3’
antisense	5’-GGTAGCCAGCATAGCGGATG-3’
probe	5’-TGGCAAGCACGACTTCCGGGTG-3’
human GAPDH	
sense	5’-CCCATGTTCGTCATGGGTGT-3’
antisense	5’-TGGTCATGAGTCCTTCCACGATA-3’
probe	5’-CTGCACCACCAACTGCTTAGCACCC-3’
human IL-8	
sense	5’-GCCGTGGCTCTCTTGGC-3’
antisense	5’-TAGCACTCCTTGGCAAAACTGC-3’
probe	5’-CTTCCTGATTTCTGCAGCTCTGTGTGAAGG-3’
human TNF-α	
sense	5’-CAAGCCTGTAGCCCATGTTG-3’
antisense	5’-GGTTGACCTTGGTCTGGTAGG-3’
probe	5’-CAAGGCTGCCCCTCCACCCA-3’
human SIRT1	
sense	5'-TCAGTGGCTGGAACAGTGAG-3'
antisense	5'-AGCGCCATGGAAAATGTAAC-3'

human β-tubulin	
sense	5'-CCAAGTTCTGGGAGGTGATCA-3'
antisense	5'-ACGCCAAGAAACAGTGATGCT-3'
murine CXCL-1	
sense	5'-CTTGAAGGTGTTGCCCTCAG-3'
antisense	5'- GTCAGAAGCCAGCGTTCAC-3'
probe	5'- AAGACAGACTGCTCTGATGGCACCG-3'
murine iNOS	
sense	5'-CAGCTGGGCTGTACAAACCTT-3'
antisense	5'- CATTGGAAGTGAAGCGTTTCG-3'
probe	5'- CGGGCAGCCTGTGAGACCTTTGA-3'
murine TNF-α	
sense	5'-CATCTTCTCAAAATTCGAGTGACAA-3'
antisense	5'- TGGGAGTAGACAAGGTACAACCC-3'
probe	5'- CACGTCGTAGCAAACCACCAAGTGGA-3'
murine GAPDH	
sense	5'- TTCACCACCATGGAGAAGGC-3'
antisense	5'- GGCATGGACTGTGGTCATGA-3'
probe	5'- TGCATCCTGCACCACCAACTGCTTAG-3'
firefly luciferase	
sense	5'- AAAAAGTTGCGCGGAGGAG -3'
antisense	5'-TTTTTCTTGCGTCGAGTTTTCC -3'
probe	5'-TGTGTTTGTGGACGAAGTACCGAAAGGTCTTAC-3'

Analysis of resveratrol effects on the human iNOS, IL-8, or TNF-α promoter activity and artificial GAS- or NF-κB-dependent promoters - To analyze the effect of resveratrol on human iNOS promoter activity, DLD-1-16kb iNOS promoter cells (5) stably transfected with a construct containing a 16 kb fragment of the human iNOS promoter cloned in front of a luciferase reporter gene were used. To analyze the effect of resveratrol on the activity of the human IL-8-, TNF-α–, GAS-, or NF-κB-dependent artificial promoter, DLD-1 cells were stably co-transfected with the plasmids pGL3-TNF-α-prom (2), pUHC13-IL8-prom (3), or the pathway detection plasmids pGAS-Luc or pNF-κB-Luc (containing 5 consensus binding sequences of the transcription factors STAT-1α or NF-κB in front of a minimal promoter) and psiRNAhH1-GFPzeo. The obtained cell pools were selected with Zeocin and analyzed for GFP and luciferase expression. Sixteen hours before cytokine-induction DLD-1-16kb iNOS-, IL-8-, TNF-α-, GAS-, or NF-κB- promoter cells were washed with PBS and incubated with

DMEM containing 2 mM L-glutamine in the absence of FCS and phenol red. Cells were pre-incubated with the indicated concentrations of resveratrol for 1h. Afterwards CM was added for additional 6h. The cells were lyzed in 1x Passive Lysis Buffer provided by the Dual-Luciferase-Reporter-Assay-System, and firefly luciferase activity as well as protein concentrations were determined. The light units of the firefly luciferase were normalized to the protein content of the extracts.

Analysis of resveratrol effects on 3'-UTR activity - To analyze the effects of resveratrol on the human iNOS-3'-UTR and SHIP-3'-UTR, transient transfections of DLD-1 cells were performed. DLD-1 cells were plated onto 24 well plates and lipofection was performed with GeneJuice according to the manufacturer's recommendations. 0.6 µg of the plasmid containing the firefly luciferase reporter gene (pcDNA4TOLuc, pcDNA4TOLuc3'UTR(iNOS), pMIR-report, or pMIR-SHIP1) were combined with 0.2 µg of the renilla reporter gene plasmid pRL-EF-1α. After overnight incubation, cells were pre-treated with different concentrations of resveratrol and stimulated for corresponding time points with CM. The cells were lyzed in 1x Passive Lysis Buffer, and firefly and renilla luciferase activities were determined in 20 µl extracts. The light units of the firefly luciferase were normalized by those of the Renilla luciferase after subtraction of extract background.

siRNA-mediated downregulation of SIRT1 expression - DLD-1 cells were transiently transfected with an anti-SIRT1 siRNA shown to down-regulate SIRT1 expression or a negative control siRNA by lipofection with HiPerFect HTS Reagent according to the manufacturer's recommendations. After 72h cells were treated as described above to analyze cytokine-induced iNOS and SIRT1 mRNA and protein expression.

Establishment of cell lines expressing an anti-KSRP shRNAs or a mutant KSRP (T692A) protein by lentiviral transduction - To generate plasmids containing a human H1 promoter driven shRNA expression cassette, enabling the intracellular expression of shRNAs against KSRP and luciferase as control, the plasmid psiRNAhH1-GFPzeo was digested with Bbs I. Into this vector double stranded oligonucleotides with matching 5'-protruding ends were ligated, generating the plasmids psiRNAhH1-GFPzeo-shLuc or psiRNAhH1-GFPzeo-shKSRP. The following oligonucleotides were used:

shKSRP	ACCTC**GATCAACCGGAGAGCAAGA**TATCGAT**TCTTGCTCTCCGGTTGATC**TTTTTG
shLuc	ACCTC**AACGTACGCGGAATACTTCGAT**TATCGATG**AATCGAAGTATTCCGCGTACGT**TTTTTTG

(siRNA sequences are bold)

The plasmids psiRNAhH1-GFPzeo-shLuc or psiRNAhH1-GFPzeo-shKSRP were restricted with Spe I and Xba I to obtain fragments containing the human H1 promoter in front of the shRNA cassette and a RNA polymerase III stop signal. These fragments were cloned into the lentiviral expression vector pCDH-CMV-MCS-EF1-copGFP restricted with Spe I and Xba I to obtain pCDH-EF1-copGFP-siLuc and pCDH-EF1-copGFP-siKSRP.

To obtain a vector control for the lentiviral pGIPZ-hmr-siKSRP vector coding for microRNA-adapted shRNA directed against human, murine, and rat KSRP, the DNA was digested with Mlu I and Xho I and treated with Klenow enzyme resulting in pGIPZ-CO.

To generate an eukaryotic expression vector coding for an EGFP-labeled mutated KSRP protein with threonine at position 692 changed to alanine, the plasmid pCMV-TAG2B-KSRP(T692A) was restricted with SRFI and Xho I and treated with Klenow enzyme. The fragment encoding the mutated KSRP was cloned into pEGFP-C1 restricted with Sma I to generate pEGFP-KSRP(T692A). Then pEGFP-KSRP(T692A) was restricted with Xba I, treated with Klenow enzyme, and restricted with Nhe I. The fragment coding for the EGFP-KSRP(T692A) was cloned into pCDH-CMV-MCS-EF1-Puro restricted with BamHI, treated with Klenow enzyme, and restricted with Nhe I resulting in the plasmid pCDH-Puro-EGFP-KSRP(T692A).

DNA sequences of all clones were controlled using the dideoxy chain termination method (Genterprise, Mainz, Germany).

For virus production, HEK 293T cells were plated at 3.5 × 10^6 cells/9 ml into cell culture dishes (10 cm in diameter). On the next day, the culture medium was exchanged 2h prior to transfection. Then cells were co-transfected with pCMVΔR8.91 (10 µg; harbors gag, pol, rev, and tat genes derived from HIV-1 (6)), pMD.G2 (4 µg; expressing the vesicular stomatitis glycoprotein G-encoding (7)), and the plasmid vectors pCDH-EF1-copGFP-siLuc, pCDH-EF1-copGFP-siKSRP, pCDH-Puro-EGFP-KSRP(T692A), pCDH-CMV-MCS-EF1-Puro, pGIPZ-hmr-siKSRP, or pGIPZ-CO (13.2 µg) using the calcium chloride transfection procedure in the presence of chloroquine sulfate (10 µM). The supernatant was removed 16h later, and 5 ml of fresh culture medium was added. Supernatants containing lentiviral particles, harvested both 24h and 48h later, were pooled and stored at −70 °C.

For transduction of DLD-1 cells with the shRNA, EGFP, or EGFP-KSRP(T692A) encoding lentiviral particles, DLD-1 cells were plated at 2 × 10^5 cells/2 ml medium per well (six well tissue culture plate). Cells were transduced on days 1 and 2 applying each 2 ml of lentivirus-containing supernatant in the presence of polybrene (8 µg/ml) (8) in a total volume of 2 ml. One day after the second round of transduction, cells were washed twice. As all lentiviral expression constructs encode for a GFP variant, successful transduction of the cells was tested by FACS analyses 7 days after the second round of transduction.

Isolation of PBMC from human blood and preparation of cell lysates – Human peripheral blood mononuclear cells (PBMC) were freshly isolated from leukocyte concentrates of human healthy donors obtained from the Blood Center at Tuebingen University Hospital, Germany. In brief, venous blood was taken from healthy adult volunteers and subjected to centrifugation at 4.000 × g for 20 min at 20°C for preparation of leukocyte concentrates. PBMC were promptly isolated by dextran sedimentation and centrifugation on Nycoprep cushions (PAA Laboratories, Linz, Austria). After washing in PBS pH 7.4, PBMC were lyzed in lysis buffer (1ml/g drained cell pellet) (50 mM Hepes pH 7.4, 200 mM NaCl, 1 mM EDTA, 1% Triton® X-100, 50 µl P8340 protease inhibitor cocktail), following 3 × 10 sec sonification. Lysates were centrifuged (15.000 × g, 1h, 4 °C) to remove cell debris and the supernatants were used for fishing experiments.

***Synthesis of ethyl 4-{4-[(1E)-2-(3,5-dihydroxyphenyl)-ethenyl]phenoxy}butanoat* -** To a dry three-neck flask resveratrol (1.02 g, 4.5 mmol), dry acetone (20 mL), potassium carbonate (1.24 g, 9 mmol), and ethyl 4-bromobutanoate (0.88 g, 4.5 mmol) were added. The reaction mixture was stirred at 50-60°C for twelve hours in reflux under argon atmosphere. After removing the solvent, the reaction mixture was separated by column chromatography using toluene/ethyl acetate (8:2) to give the desired compound (0.07 g, 5%).

The product was recrystallized from ethanol (40%). Mp 124-125°C. ^1H NMR (CD_3OD, ppm) δ 7.45 (d, 2H, J = 8.7 Hz, Ar-H), 7.10-6.85 (m, 4H, Ar-H and olefinic H), 6.52-6.46 (m, 2H, Ar-H), 6.24-6.14 (m, 1H, Ar-H), 4.17 (q, 2H, J = 7.1 Hz, OCH_2), 4.06 (t, 2H, J = 6.1 Hz, OCH_2), 2.54 (t, 2H, J = 6.1 Hz, CH_2), 2.10 (quint, 2H, J = 6.1 Hz, CH_2), 1.27 (t, 3H, J = 7.1 Hz, CH_3). ^{13}C NMR (CD_3OD, ppm) δ 175.5 160.4, 160.1, 141.6, 132.1, 129.5, 129.1, 128.3, 116.1, 106.3, 103.2, 68.4, 62.0, 32.2, 26.3, 14.9. MS: m/z = 342 (M^+, 10), 297 (6), 279 (1), 228 (4), 115 (100), 87 (94). CHN calc. x 0.25 H_2O: C 69.25, H 6.54; found: C 68.99, H 6.33.

***Synthesis of 4-{4-[(1E)-2-(3,5-dihydroxyphenyl)-ethenyl]phenoxy}butyric acid* -** To a round-bottomed flask the above synthesized ester (0.09 g, 0.26 mmol), methanol (3 mL), water (0.1 g), and potassium hydroxide (0.6 g, 10.7 mmol) were added. The resulting mixture was stirred for two days at room temperature. After this, the mixture was acidified using 6N HCl. After removing the solvent, the residue was extracted three times with ethyl acetate. The compound was isolated by column chromatography using toluene/ethyl acetate/glacial acetic acid (8:2:0.1) to give the desired acid (0.05 g, 60 %). Mp 212-215°C. ^1H NMR (CD_3OD, ppm) δ 7.46 (d, 2H, J = 8.8 Hz, Ar-H), 7.11-6.88 (m, 4H, Ar-H and olefinic H), 6.51-6.47 (m, 2H, Ar-H), 6.25-6.17 (m, 1H, Ar-H), 4.06 (t, 2H, J = 6.2 Hz, OCH_2), 2.53 (t, 2H, J = 6.2 Hz, CH_2), 2.10 (quint, 2H, J = 6.2 Hz, CH_2). ^{13}C NMR (CD_3OD, ppm) δ 174.0, 160.5, 160.1, 141.6, 132.2, 129.5, 129.1, 128.2, 116.1, 106.3, 103.2, 68.5, 31.9, 26.3. MS: m/z = 314 (M^+, 36), 228 (100), 211 (12), 181 (18), 152 (11). CHN calc.: C 68.76, H 5.77; found: C 68.37, H 5.90.

***Immobilization of resveratrol* -** For immobilization of the ligands, 16.7 mg resveratrol probe (4-{4-[(1E)-2-(3,5-dihydroxyphenyl)-ethenyl]phenoxy}butyric acid) and 9.58 mg phenoxybutyric acid (PBA, negative control) were first solved in 1 ml dioxane (= ligand solution). Then, suspensions of 200 µl Toyopearl® AF 650 Amino beads were washed with water, dioxane 50% (v/v), and pure dioxane on a G4 glass filter crucible. The ligand solutions (150 µmol in 1 ml solvent) were mixed with 10 µl 1 N HCl, 1.5 mmol N,N-dicyclohexylcarbidiimide (DCC), and the prewashed beads. After 72h at RT on a rotator, the beads were washed with solvent and afterwards with acetate buffer (0.1 M sodium acetate, 0.1 M acetic acid, pH 4, 0.5 M NaCl) and Tris buffer (0.1 M Tris-HCl pH 8, 0.5 M NaCl), alternately, to remove ionic-bound ligand. The beads were finally washed with 20% (v/v) ethanol.

NMR spectroscopy and mass spectrometry - ^1H and ^{13}C NMR spectra of the compounds were recorded on a Bruker Advance DPx200 (200 and 50 MHz). Chemical shifts are reported in d units (ppm) relative to Me4Si line as internal standard (s, br s, d, m, Cq for singlet, broad singlet, doublet, multiplet and quaternary carbon, respectively) and J values are reported in Hertz. Mass spectra (MS)

were obtained with a Shimadzu (GC-17A; MS-QP5050A) spectrometer. Purity of the compounds was established by combustion analysis with a Perkin-Elmer 2400 CHN elemental analyzer.

Protein-fishing assays **-** For protein-fishing assays, 50 µl beads (corresponding to 100 µl of 50% (v/v) bead slurry) were incubated with the cell lysate (500 mg) for 4h at 4 °C on a rotator in 750 µl binding buffer. Beads were extensively washed 3 times (in 500 µl binding buffer) by centrifugation (5000 × g, 3 min, 4 °C). Bound proteins were eluted by addition of 50 µl 4 M urea, pH 8.5 to 50 µl beads during a 30 min incubation at 4 °C. Eluted proteins were separated by centrifugation (7000 × g, 5 min, 4 °C) and subjected to 2D-DIGE analysis. For silver staining analysis, eluted proteins were mixed with 50 µl SDS-PAGE loading buffer (20 mM Tris pH 8, 2 mM EDTA, 0.5% (w/v) SDS, 10% (v/v) 2-mercaptoethanol) and boiled for 5 min at 96 °C.

2D-Gel electrophoresis and Ettan-DIGE Analysis **–** For 2D-DIGE analysis, eluted proteins were labeled with the CyDyes^{TM} Cy3 and Cy5. The CyDye^{TM} (solubilized in water-free DMF) was added to the samples (1 µl of the 0.8 mM working solution for 10 µg protein) and incubated for 30 min at 4 °C under light protection. The reaction was stopped by addition of 1 µl 10 mM lysine.

Isoelectric focusing was performed on 17 cm immobilized pH gradient (IPG) strips with a non-linear pH gradient (pH 3 to pH 10; Ready Strip^{TM}). Proteins were diluted with PCT buffer (8 M urea, 2 M thiourea, 4% (w/v) chaps, ampholyte pH 3-10 0.7% (v/v), dithiothreitol (DTT) 65 mM, 10 µg/ml bromophenol blue). Then, 50 µl P8340 protease inhibitor cocktail per gram protein was added and 300 µl sample solution containing up to 300 µg labeled proteins was pipetted into a 17 cm focusing tray. An IPG strip was placed on the sample to rehydrate with the sample solution for 12h at 50 V and 10 °C. After rehydration, electrode wicks were moistened with 5 µl PCT-buffer and placed on the electrodes. IPD stripes were developed with a linear voltage gradient with a final voltage of 10,000 V for total 30 kVh.

For the second dimension, a SDS-PAGE was performed. IPG-strips were equilibrated for 2 × 7 min in equilibration buffer I (6 M urea, 4% (w/v) SDS, 0.05 M Tris pH 8.8, 30% (v/v) glycerol, 1% (v/w) DTT), 2 × 7 min in equilibration buffer II (6 M urea, 4 (w/v) SDS, 0.05 M Tris pH 8.8, 30% (v/v) glycerol, 4% (w/v) iodoacetamide), and 1 × 10 sec in SDS-PAGE running buffer (200 mM glycine, 25 mM Tris, 3 mM SDS). The IPG-strips were placed on top of a 20 × 20 cm polyacrylamide gel containing 12% acrylamide. The IPG-strip was ingrained into an agarose gel (0.5% (w/v) agarose, 0.001% bromophenol blue in SDS-PAGE running buffer (25 mM Tris, 190 mM glycine, 3.5 mM SDS)). A filter paper (5 mm diameter) was moistened with 5 µl of protein markers (peqGOLD IV) and ingrained together with the IPG-strip. Gels were developed at 16 mA for the stacking gel and 24 mA for the separation gel for about 8h (BioRad PowerPac^{TM}).

DIGE pictures were obtained by an ETTAN^{TM} DIGE Imager system (GE Healthcare) and evaluated via ImageQuant^{TM} TL software. The fluorescence emission of Cy3 and Cy5 dyes were scanned in their corresponding channels at 595 and 680 nm, after excitation at 540 and 635 nm, respectively. Required exposure time was 2 sec with a resolution of 100 µm. Spots on scanned 2D-DIGE gels were detected using the DeCyder 7.0 software. DeCyder 7 algorithm was used to calculate appropriate spot values. The threshold was set as 2.0. Spots exceeding a slope of 1.0 were considered artificial.

Silver staining was performed by fixing the gels in a fixing solution (45% (v/v) methanol, 45% (v/v) H_2O, 5% (v/v) acetic acid) for 30 min. Gels were then washed and sensitized by addition of $Na_2S_2O_3$ solution (1 mg/ml). After two more washing steps with Milli-Q water, the staining solution ($AgNO_3$, 1 mg/ml) was added, two washing steps with water were performed and then the developing solution (2.5% Na_2CO_3, 0.03% formalin in H_2O) was added. The staining reaction was stopped using 1% (v/v) acetic acid. Prior further steps, gels were washed with water. The silver stained gels were acquired with a CCD camera system (CabUVis, Hitachi HV-C20M 3CCD, Sarstedt AG Co., Nümbrecht, Germany).

MS analytics of fished proteins was conducted at the Proteome Center at Univ. Tuebingen via in-gel digestion and nanoflow liquid chromatography tandem MS (nano-LC-ESI-MS/MS) on a QSTAR Pulsar hybrid qTOF mass spectrometer (AB-MDS Sciex) as described previously (9). Bioinformatic analysis of the identified peptides was subsequently performed with Matrix Science Mascot.

Immunoprecipitation-qRT-PCR assay – For the determination of intracellular protein-RNA interactions, DLD-1 cells were pre-incubated with 30 µM Resveratrol or the same volume DMSO, respectively and incubated for 4h with or without the cytokine mixture. Cells were lyzed in a buffer containing 10 mM Hepes pH 7.9, 1.5 mM $MgCl_2$, 10 mM KCl, 0.5 mM DTT, 0.1 % NP40, 0.2 % VRC, 100 U/ml RNasin and 1x complete EDTA-free protease and phosphatase inhibitor cocktail (Roche)). Cell lysates (2 mg) were preincubated with 20 µl protein A/G plus agarose beads (Santa Cruz Biotechnology, Heidelberg, Germany) in 500 µl NT2-buffer (50 mM Tris/HCl pH 7.4, 150 mM NaCl, 1 mM $MgCl_2$, 0.05 % NP-40) for 30 min at 4°C. After a centrifugation at 1200g for 5 min at 4°C, the supernatant was incubated with 50 µl protein A/G plus agarose beads (pre-coated with the specific antibody in NT2-buffer supplemented with 200 µg tRNA and 0.5 mg/ml Heparin for 16h at 4°C) for 3h at 4°C. Subsequently the beads were washed 4 times in 500 µL NT2-buffer and then digested with 0.5 mg/ml Proteinase K in NT2-buffer containing 0.1 % SDS for 15 min at 55°C. Then 1 ng/sample *in-vitro* transcribed luciferase RNA was added to normalize for subsequent purification steps. RNA was isolated using the RNeasy Mini Kit (Qiagen, Hilden, Germany) as described by the manufacturer. The amount of IL-8, iNOS, and TNF-α mRNA bound by KSRP was determined by qRT-PCR using the primers and probes described above. The C(T)-values for IL-8, iNOS, and TNF-α mRNA were normalized to the C(T)-values of luciferase mRNA.

SUPPLEMENTARY FIGURE LEGENDS

Fig. S1: Effects of resveratrol on the human iNOS, IL-8, or TNF-α promoter and artificial NF-κB- or STAT-1α-dependent promoter activity.

DLD-1 cells stably transfected with human iNOS (**A**), IL-8 (**B**), or TNF-α (**C**) promoter firefly luciferase reporter constructs or pNF-κB-Luc (**D**) or pGAS-Luc (**E**; 5 x consensus binding sites for NF-κB or STAT-1α in front of a basal promoter cloned before the luciferase reporter gene) were pre-incubated with different amounts of resveratrol for 1h and induced with CM for additional 6h. Then, cells were

lyzed and the luciferase activity was determined. The luciferase activity (luc-activity) was normalized to the protein content of the extracts. The relative luciferase activities in CM-induced cells were set to 100%. Shown are the mean ± SEM of n = 8-10 experiments (*** = p < 0.001, * = p < 0.05, ns = not significant vs. CM-treated DLD-1 cells; one way Anova).

Fig. S2: Resveratrol downregulates iNOS-UTR-dependent luciferase expression.

DLD-1 cells were transiently transfected with pcDNA4TOLuc3'UTR (iNOS) containing the 3'-UTR of the human iNOS mRNA behind the firefly luciferase stop codon. For normalization of transfection efficiency, cells were co-transfected with pRL-EF1α. Then the cells were pre-incubated with different concentrations of resveratrol for 1h and incubated with CM for 6h. Cell extracts were prepared and the firefly and renilla luciferase activities were determined. Firefly luciferase activities were normalized to those of the renilla luciferase and the relative luciferase activities (luc-activity) in only CM-treated cells were set to 100%. Shown are the mean ± SEM of n = 8-10 experiments (* = p < 0.01, ns = not significant vs. CM-treated DLD-1 cells; one way Anova).

Fig. S3: Resveratrol-mediated inhibition of iNOS expression is SIRT1-independent.

DLD-1 cells were pre-treated with the SIRT1 inhibitor sirtinol (**Sirtinol**, 30 µM), a specific anti-SIRT1 siRNA (**siSIRT1**), or an unrelated control siRNA (**siCo**). Then the cells were incubated with resveratrol (**Res**; 30 µM) for 1h and treated with CM for additional 6h. RNA and proteins were isolated and analyzed for iNOS, SIRT1, GAPDH, or β-tubulin mRNA expression by qRT-PCR (**A, B, D**) or SIRT1 and GAPDH protein expression by Western blot (**C**).

A. Data shown are mean ± SEM of n = 5-6 qRT-PCR analyses. The CM-induced iNOS mRNA expression in CM-(light gray) or CM + sirtinol-(dark gray) treated DLD1 cells was set to 100% (*** = p < 0.001 vs. CM-treated DLD-1 cells; $ = p < 0.05 vs. CM + sirtinol-treated DLD-1 cells; one way Anova).

B. Data shown are mean ± SEM of n = 4-5 qRT-PCR analyses. The SIRT1 mRNA expression in CM + siCo-(light gray) or CM + siCo + Res-(dark gray) treated DLD1 cells was set to 100% (*** = p < 0.001, ns = not significant vs. CM + siCo-treated DLD-1 cells; $$$ = p < 0.001 vs. CM + siCo + Res-treated DLD-1 cells; one way Anova).

C. Shown is one (of three) representative Western blots analyzing SIRT1 protein expression (**SIRT1**) in CM + Res-treated DLD-1 cells incubated with an unrelated (**siCo**) or a specific anti-SIRT1 siRNA (**siSIRT1**) using a specific anti-SIRT1 antibody. SIRT1 protein (**SIRT1**) expression was normalized by analyzing the expression of GAPDH protein (**GAPDH**) in the same extracts.

D. Data shown are mean ± SEM of n = 5-6 qRT-PCR analyses. The CM-induced iNOS mRNA expression in CM + siCo-(light gray) or CM + siSIRT1-(dark gray) treated DLD1 cells was set to 100% (*** = p < 0.001, ** = p < 0.01 vs. CM + siCo-treated DLD-1 cells; $ = p < 0.05 vs. CM + siSIRT1-treated DLD-1 cells; one way Anova).

Fig. S4: Identification of KSRP as a direct binding target of resveratrol.

(**A**) Chemical structures of (I) resveratrol, (II) γ-carboxybutyl-resveratrol and (III) γ- phenoxybutyric acid for immobilization. (**B**) (I) Resveratrol and (II) phenoxybutyric acid (negative control) linked to an insoluble matrix (beads) for fishing experiments. (**C**) 2D-DIGE of resveratrol- and phenoxybutyric acid-fished proteins from PBMC lysates. Red: Cy5 labeled proteins fished by resveratrol, green: Cy3 labeled proteins fished by phenoxybutyric acid, yellow: merge of Cy5 and Cy3 labeled proteins.

Fig. S5: Resveratrol reduces the pro-inflammatory mRNA expression in a KSRP-dependent manner.

DLD-1 cells were stably transfected with pCDH-EF1-copGFP-siKSRP (**siKSRP**), pCDH-EF1-copGFP-siLuc (**siLuc**), pGIPZ-hmr-siKSRP (**si**), or pGIPZ-CO (**CO**) resulting in the intracellular expression of KSRP siRNA (**siKSRP** or **si**), a Luc siRNA (**siLuc**), or no siRNA (**CO**, control).

A DLD-1 **siLuc** or **siKSRP** cells were treated with CM for 6h. RNA was isolated and analyzed for iNOS and GAPDH (for normalization) mRNA expression by qRT-PCR. Data shown are mean ± SEM of n = 4-5 qRT-PCR analyses. The CM-induced mRNA expression in **siLuc** cells was set to 100% (*** = $p < 0.001$ vs. CM treated DLD-1 **siLuc** cells; one way Anova).

B + C DLD-1 **CO** or **si** cells were treated with CM for 2h. RNA was isolated and analyzed for IL-8 (**B**), TNF-α (**C**), and GAPDH (for normalization) mRNA expression by qRT-PCR. Data shown are mean ± SEM of n = 4-5 qRT-PCR analyses. The CM-induced mRNA expression in **CO** cells was set to 100% (*** = $p < 0.001$ vs. CM-treated DLD-1 **CO** cells; one way Anova).

D DLD-1 **siLuc** or **siKSRP** cells were pre-incubated with resveratrol (**Res**; 30 µM) for 1h and then treated with CM for additional 6h. RNA was isolated and analyzed for iNOS and GAPDH (for normalization) mRNA expression by qRT-PCR. Data shown are mean ± SEM of n = 4-5 qRT-PCR analyses. The CM-induced mRNA expression in **siLuc** or **siKSRP** cells was set to 100% (*** = $p < 0.001$ vs. CM treated DLD-1 **siLuc** cells; $$$ = $p < 0.001$, $ns_{\$}$ not significant vs. CM-treated DLD-1 **siKSRP** cells; one way Anova).

E + F DLD-1 **CO** or **si** cells were pre-incubated with resveratrol (**Res**; 30 µM) for 1h and then treated with CM for additional 2h. RNA was isolated and analyzed for IL-8 (**E**), TNF-α (**F**), and GAPDH (for normalization) mRNA expression by qRT-PCR. Data shown are mean ± SEM of n = 4-5 qRT-PCR analyses. The CM-induced mRNA expression in **CO** or **si** cells was set to 100% (*** = $p < 0.001$ vs. CM-treated DLD-1 **CO** cells; $$$ = $p < 0.001$, $ns_{\$}$ not significant vs. CM-treated DLD-1 **si** cells; one way Anova).

Fig. S6: Resveratrol does not change KSRP protein levels.

DLD-1 cells were pre-treated with 30 µM resveratrol for 1h. CM was added and after 6h protein extracts were isolated and KSRP levels were determined by Western blot using a specific anti-KSRP antibody (**KSRP**). For normalization, the expression of β-tubulin (**β-tubulin**) was analyzed in parallel using a specific anti-β-tubulin antibody. Shown is one (of four) representative Western blots.

Fig. S7: Inhibition of p38 MAPK by SB reduces, whereas inhibition of ATM-kinase by KU or PI3K-AKT by wortmannin enhances cytokine induced iNOS expression in human DLD-1 cells.

DLD-1 cells were pre-incubated with the p38 MAPK inhibitor **SB**203580 (1 to 10 µM), the ATM kinase inhibitor **KU**55933 (10 or 30 µM), or the PI3K-AKT inhibitor wortmannin (**WM**; 3 or 10 µM) for 1h and then treated with a cytokine mixture (**CM**) for additional 6h. RNA was isolated and analyzed for iNOS and GAPDH (for normalization) mRNA expression by qRT-PCR. Data shown are mean ± SEM of n = 4-6 qRT-PCR analyses. The CM-induced mRNA expression was set to 100% (*** = p < 0.001, ** = p < 0.01 vs. CM-treated DLD-1 cells; one way Anova).

Fig. S8: Overexpression of the mutant KSRP(T692A) isoform in DLD-1 cells.

To analyze whether mutation of threonine 692 to alanine modifies the effect of resveratrol on pro-inflammatory gene expression, we created lentiviral transduced DLD-1 cells expressing an EGFP-KSRP(T692A) fusion protein (**TA** cells) or **EGFP** (as control). Expression of KSRP and the EGFP-KSRP(T692A) fusion protein in **EGFP** and **TA** cells was analyzed by Western blot experiments using a specific anti-KSRP antibody. For normalization, the expression of GAPDH was analyzed in parallel using a specific anti-GAPDH antibody. Shown is one of four representative Western blots. The postions of the EGFP-KSRP(T692A) fusion protein, KSRP and GAPDH are indicated.

Fig. S9: Resveratrol enhances KSRP-exosome interaction.

DLD-1 cells were pre-treated with or without 30 µM resveratrol (**Res**) and incubated with CM for 15min. Cell lysates were prepared and subjected to immunoprecipitation using a polyclonal anti-KSRP (α**KSRP**) antibody or total **IgG**. The immunoprecipated material was separated by SDS-PAGE and the amounts of total **KSRP** protein (**A**) and of the exosomal component **PM-Scl** (**B**) were analyzed by Western blot experiments using specific antibodies. In the upper panel one representative out of four co-immunoprecipitation assays is shown. In the lower panel the summary of densitometric analyses of all Western blots is shown (*= p < 0.05 vs. CM-induced cells not treated with resveratrol; t test)

REFERENCES TO SUPPLEMENTAL DATA

1. O'Connell, R.M., Chaudhuri, A.A., Rao, D.S. and Baltimore, D. (2009) Inositol phosphatase SHIP1 is a primary target of miR-155. *Proc Natl Acad Sci U S A*, **106**, 7113-7118.
2. Rether, J., Erkel, G., Anke, T. and Sterner, O. (2004) Inhibition of inducible TNF-alpha expression by oxaspirodion, a novel spiro-compound from the ascomycete Chaetomium subspirale. *Biol Chem*, **385**, 829-834.
3. Holtmann, H., Winzen, R., Holland, P., Eickemeier, S., Hoffmann, E., Wallach, D., Malinin, N.L., Cooper, J.A., Resch, K. and Kracht, M. (1999) Induction of interleukin-8 synthesis integrates effects on transcription and mRNA degradation from at least three different cytokine- or stress-activated signal transduction pathways. *Mol Cell Biol*, **19**, 6742-6753.
4. Briata, P., Forcales, S.V., Ponassi, M., Corte, G., Chen, C.Y., Karin, M., Puri, P.L. and Gherzi, R. (2005) p38-dependent phosphorylation of the mRNA decay-promoting factor KSRP controls the stability of select myogenic transcripts. *Mol Cell*, **20**, 891-903.
5. Fechir, M., Linker, K., Pautz, A., Hubrich, T., Forstermann, U., Rodriguez-Pascual, F. and Kleinert, H. (2005) Tristetraprolin regulates the expression of the human inducible nitric-oxide synthase gene. *Mol Pharmacol*, **67**, 2148-2161.

6. Zufferey, R., Nagy, D., Mandel, R.J., Naldini, L. and Trono, D. (1997) Multiply attenuated lentiviral vector achieves efficient gene delivery in vivo. *Nat Biotechnol*, **15**, 871-875.
7. Follenzi, A., Sabatino, G., Lombardo, A., Boccaccio, C. and Naldini, L. (2002) Efficient gene delivery and targeted expression to hepatocytes in vivo by improved lentiviral vectors. *Hum Gene Ther*, **13**, 243-260.
8. Davis, H.E., Rosinski, M., Morgan, J.R. and Yarmush, M.L. (2004) Charged polymers modulate retrovirus transduction via membrane charge neutralization and virus aggregation. *Biophys J*, **86**, 1234-1242.
9. Hoschle, B., Gnau, V. and Jendrossek, D. (2005) Methylcrotonyl-CoA and geranyl-CoA carboxylases are involved in leucine/isovalerate utilization (Liu) and acyclic terpene utilization (Atu), and are encoded by liuB/liuD and atuC/atuF, in Pseudomonas aeruginosa. *Microbiology*, **151**, 3649-3656.

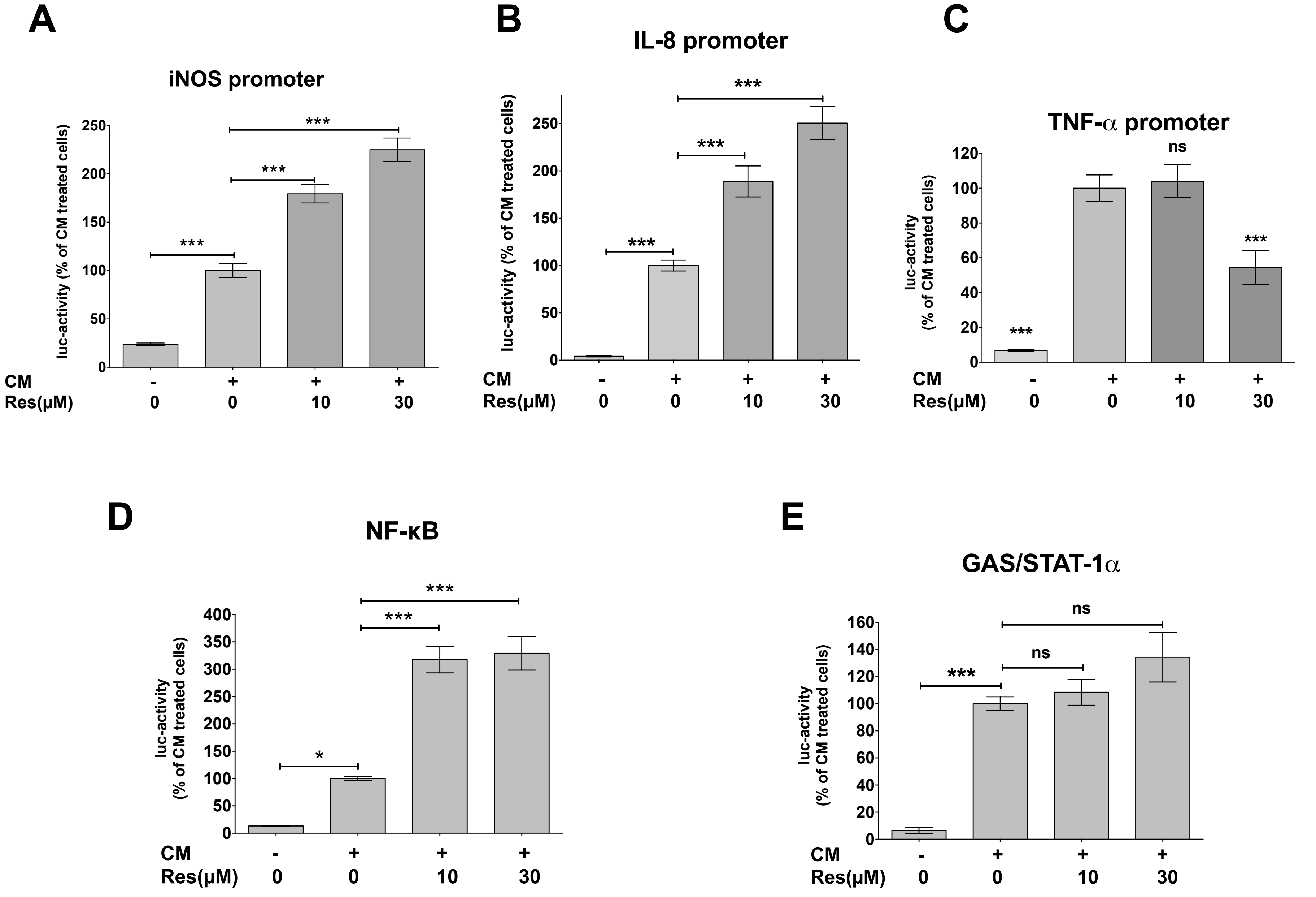
A
iNOS promoter
luc-activity (% of CM treated cells)
CM
Res(μM)
B
IL-8 promoter
C
TNF-α promoter
ns
D
NF-κB
E
GAS/STAT-1α
***
*
- + + +
0 0 10 30

Fig S1

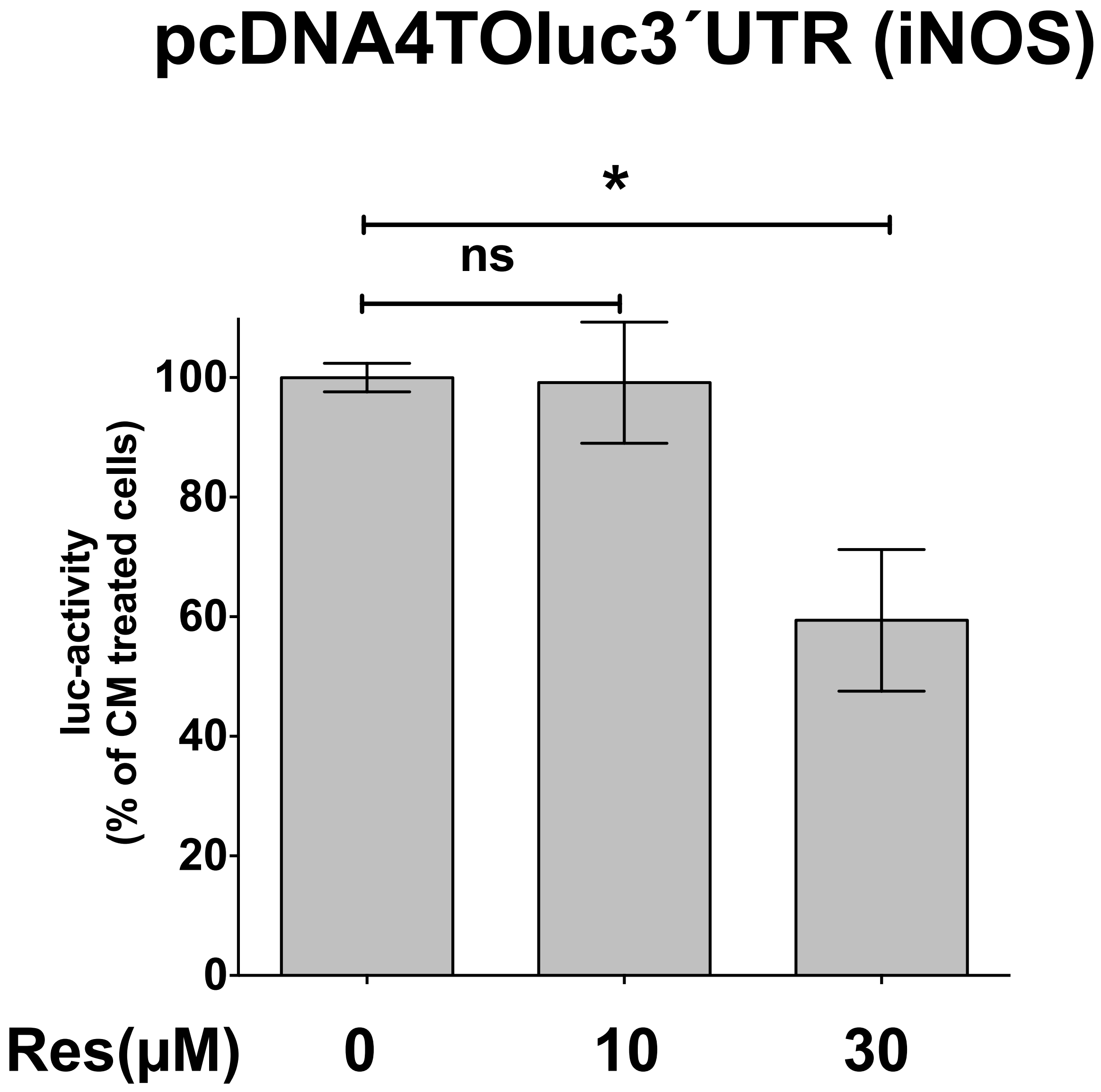
pcDNA4TOluc3´UTR (iNOS)
*
ns
luc-activity
(% of CM treated cells)
0
20
40
60
80
100
Res(µM)
0
10
30

Fig S2

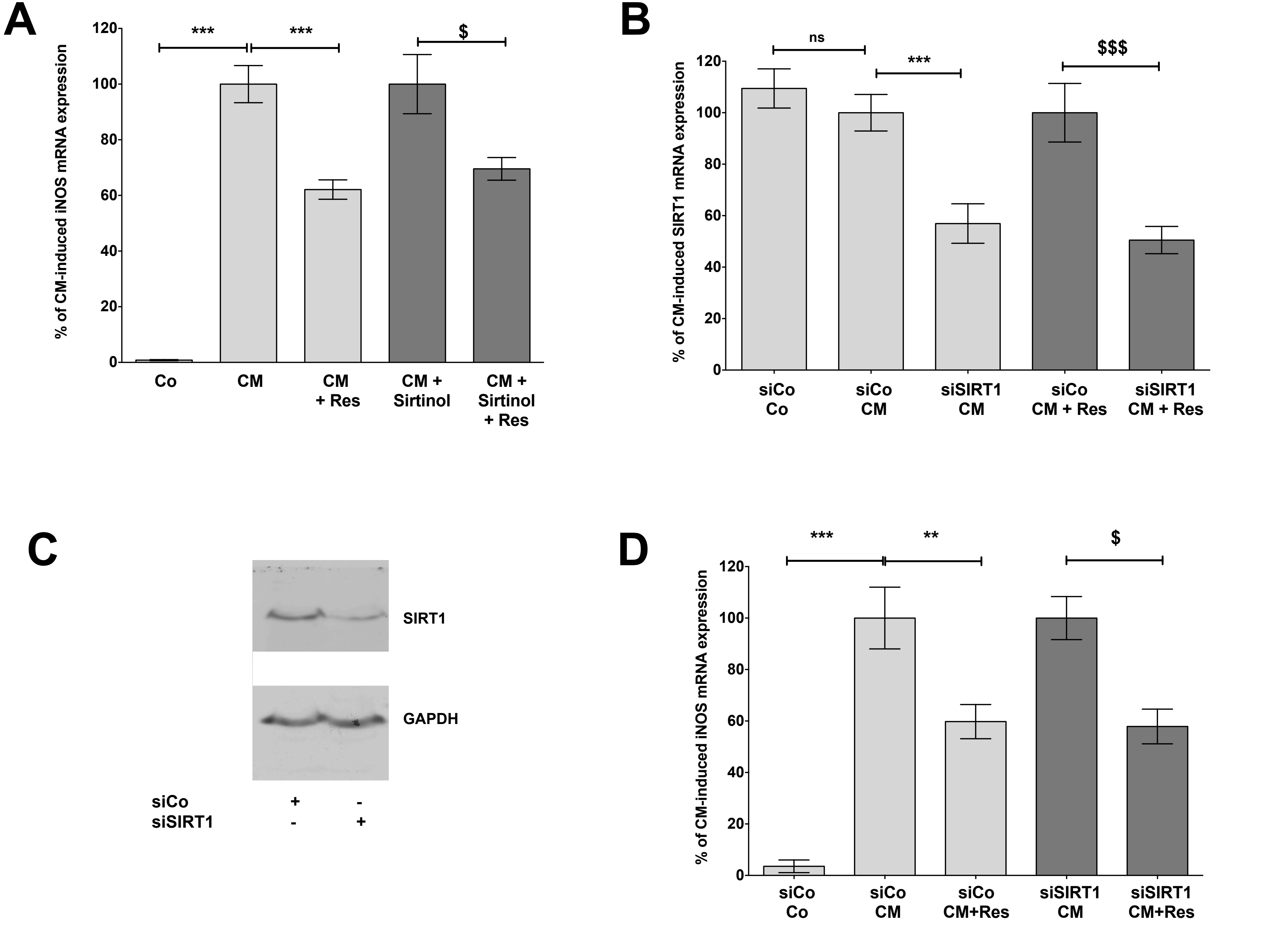
A
% of CM-induced iNOS mRNA expression
120
100
80
60
40
20
0
***
***
$
Co
CM
CM + Res
CM + Sirtinol
CM + Sirtinol + Res
B
% of CM-induced SIRT1 mRNA expression
ns
***
$$$
siCo Co
siCo CM
siSIRT1 CM
siCo CM + Res
siSIRT1 CM + Res
C
SIRT1
GAPDH
siCo + -
siSIRT1 - +
D
% of CM-induced iNOS mRNA expression
***
**
$
siCo Co
siCo CM
siCo CM+Res
siSIRT1 CM
siSIRT1 CM+Res

Fig S3

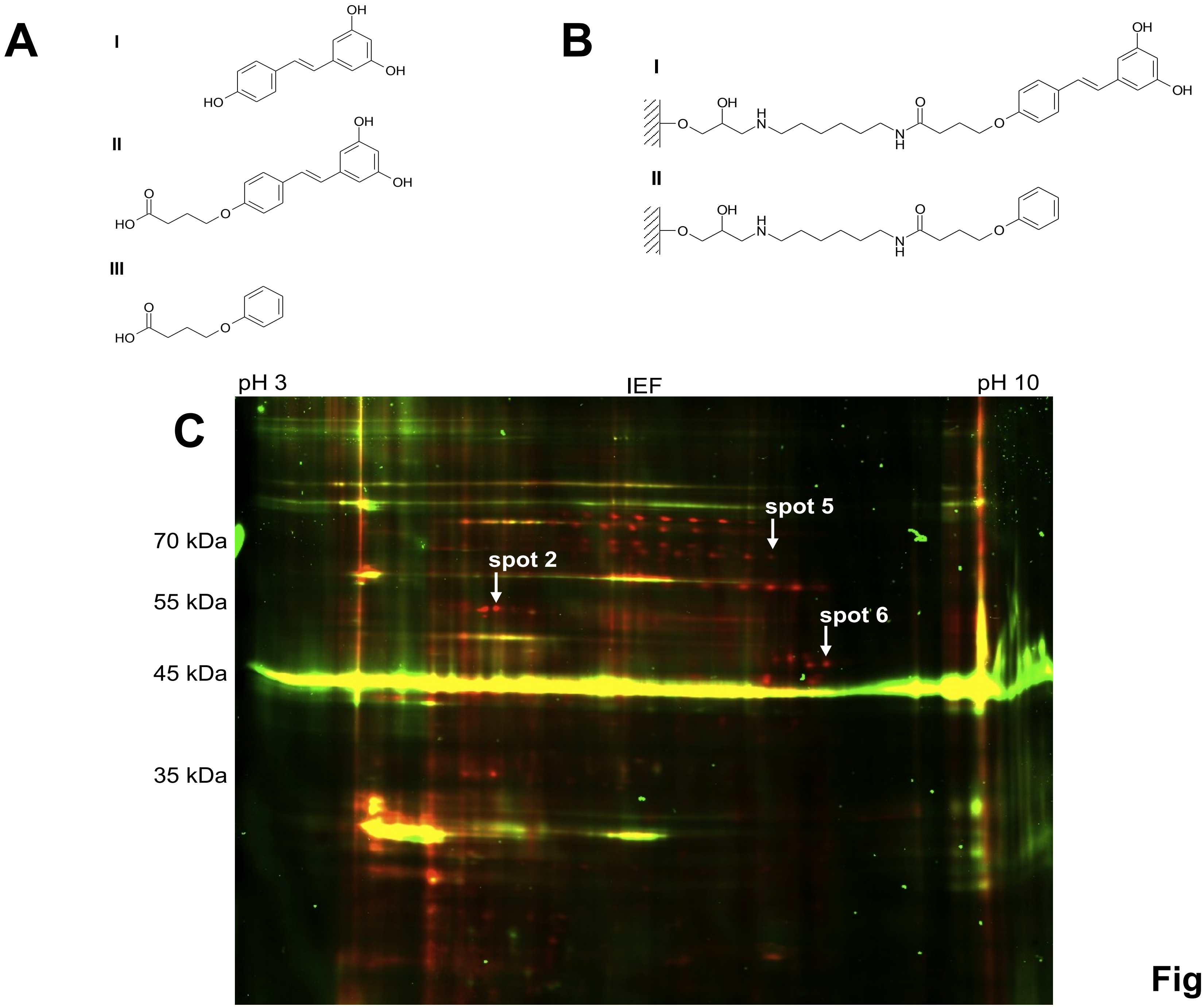
A
I
OH
HO
OH
II
OH
O
HO
O
OH
III
O
HO
O
B
I
OH
OH
H
N
O
O
N
H
O
OH
II
OH
H
N
O
O
N
H
O
C
pH 3
IEF
pH 10
70 kDa
55 kDa
45 kDa
35 kDa
spot 2
spot 5
spot 6

Fig S4

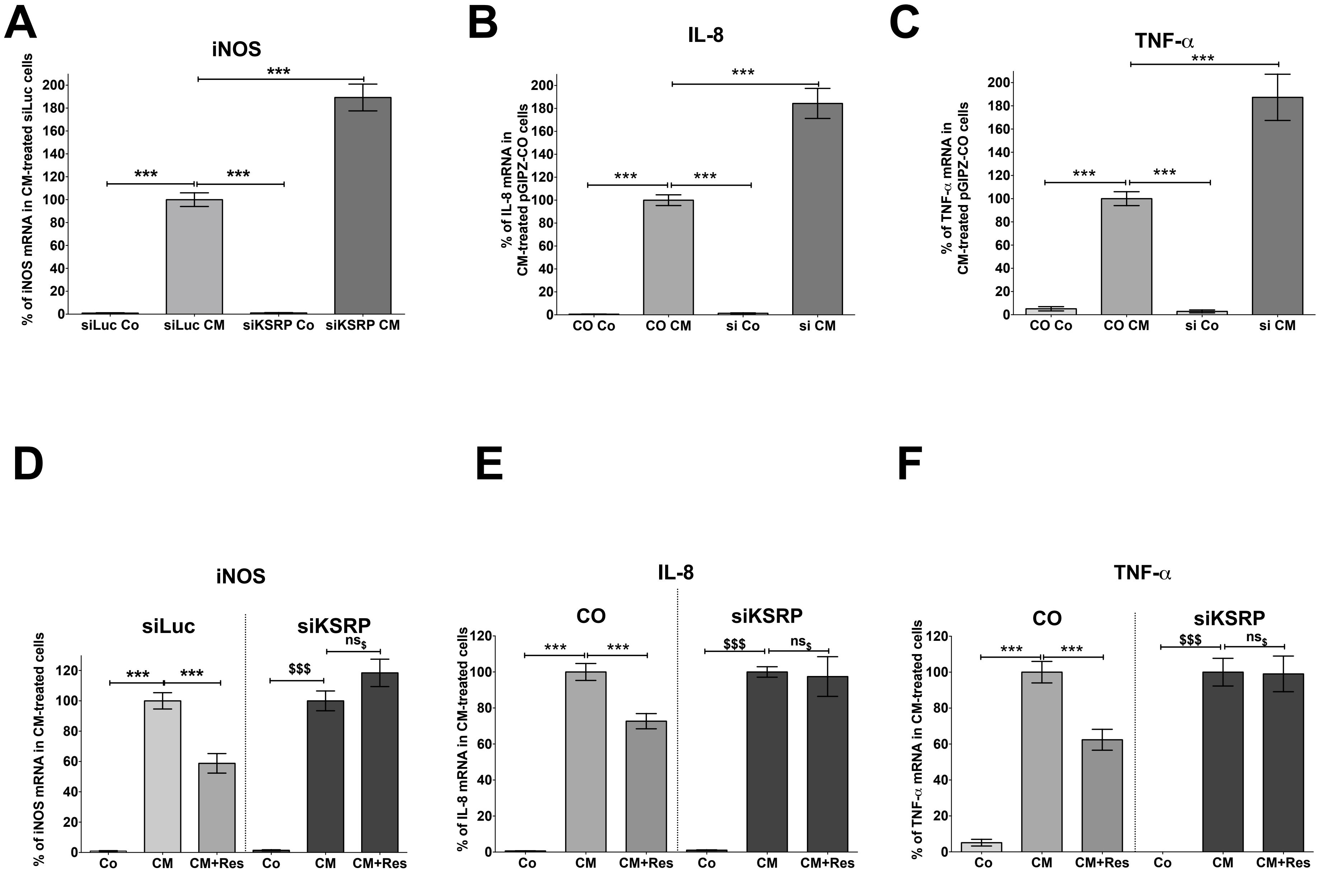
A
iNOS
% of iNOS mRNA in CM-treated siLuc cells
siLuc Co
siLuc CM
siKSRP Co
siKSRP CM
B
IL-8
% of IL-8 mRNA in CM-treated pGIPZ-CO cells
CO Co
CO CM
si Co
si CM
C
TNF-α
% of TNF-α mRNA in CM-treated pGIPZ-CO cells
D
iNOS
siLuc
siKSRP
% of iNOS mRNA in CM-treated cells
Co
CM
CM+Res
E
IL-8
CO
siKSRP
% of IL-8 mRNA in CM-treated cells
F
TNF-α
% of TNF-α mRNA in CM-treated cells

Fig S5

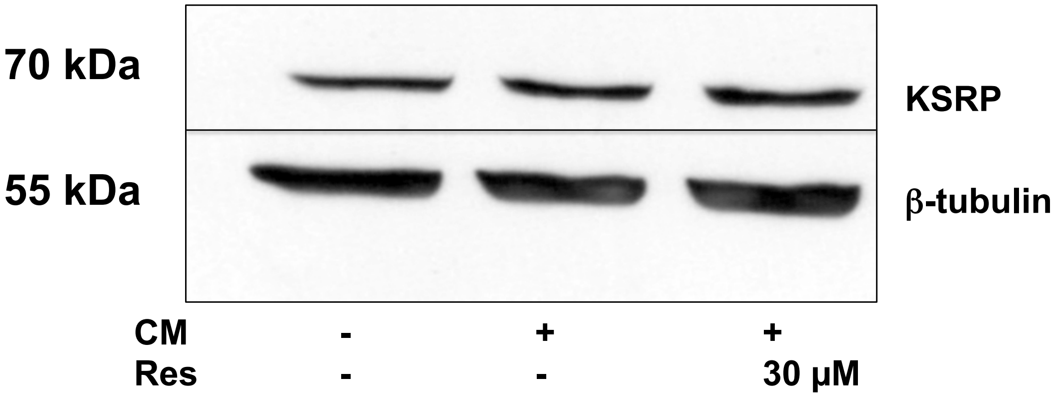

Fig S6

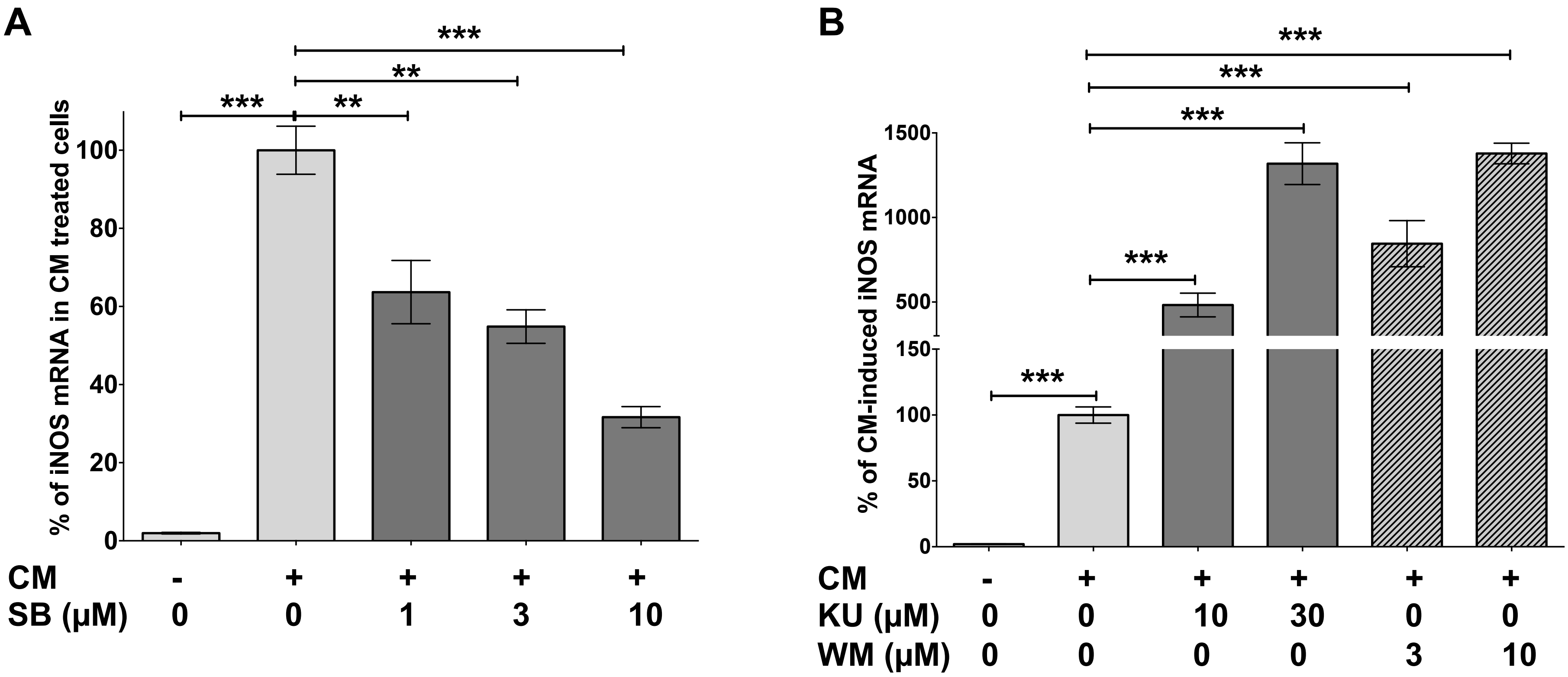
A
% of iNOS mRNA in CM treated cells
CM
SB (μM)
B
% of CM-induced iNOS mRNA
CM
KU (μM)
WM (μM)

Fig. S7

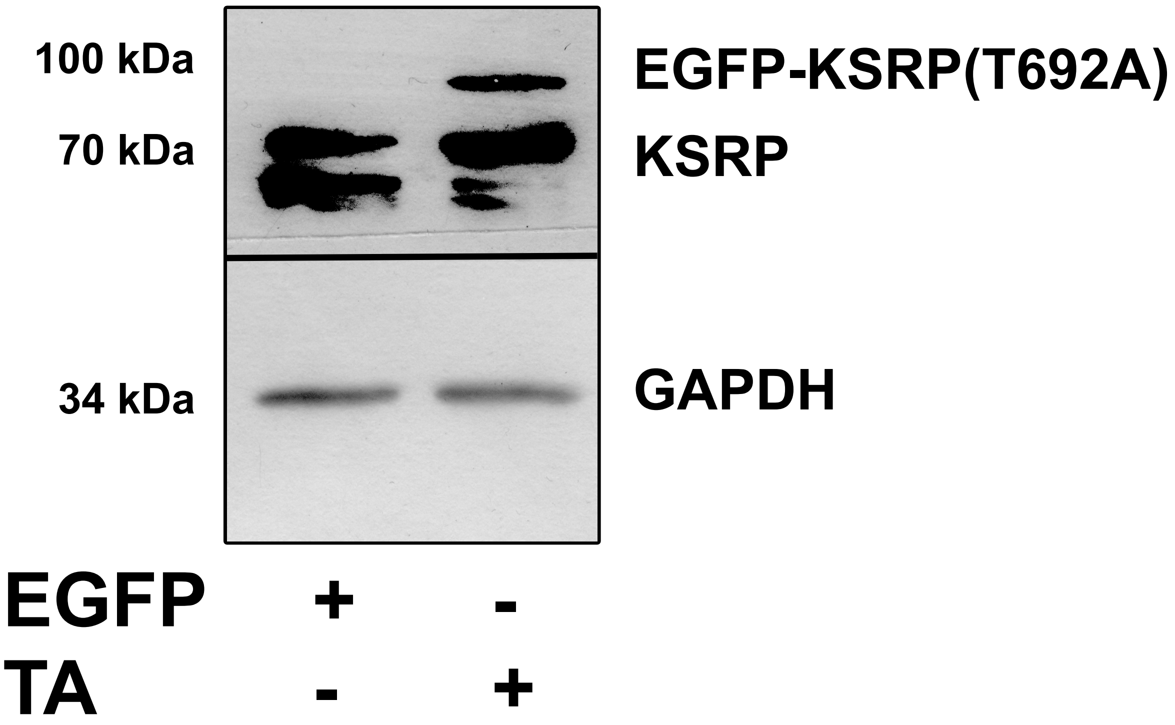

Fig. S8

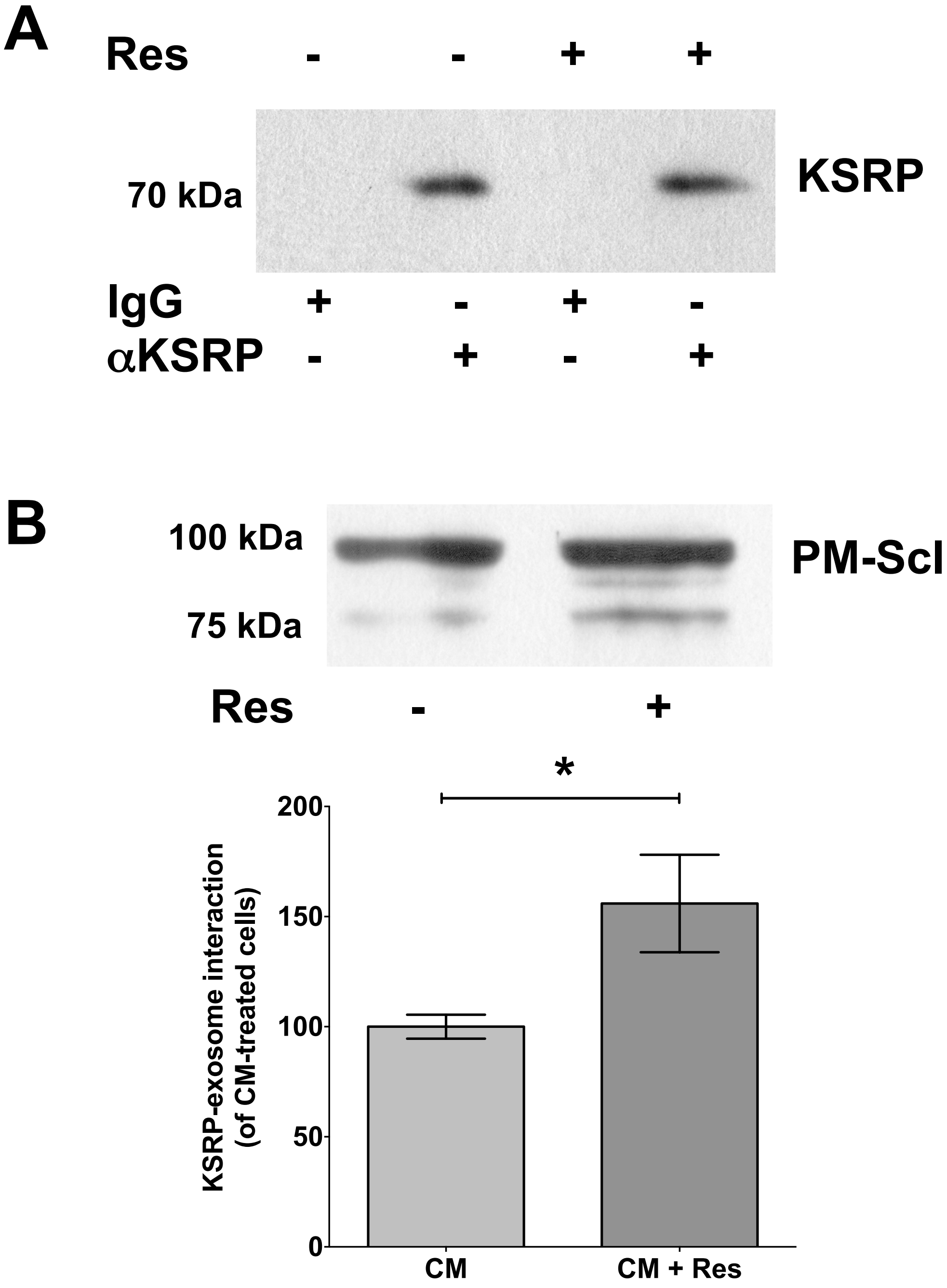
A
Res - - + +
70 kDa
KSRP
IgG + - + -
αKSRP - + - +
B
100 kDa
75 kDa
PM-Scl
Res - +
*
KSRP-exosome interaction (of CM-treated cells)
200
150
100
50
0
CM
CM + Res

Fig. S9
